# Microbial Community Composition Impacts Pathogen Iron Availability during Polymicrobial Infection

**DOI:** 10.1371/journal.ppat.1006084

**Published:** 2016-12-14

**Authors:** Apollo Stacy, Nader Abraham, Peter Jorth, Marvin Whiteley

**Affiliations:** Department of Molecular Biosciences, Institute of Cellular and Molecular Biology, John Ring LaMontagne Center for Infectious Disease, The University of Texas at Austin, Austin, TX United States of America; Northwestern University, UNITED STATES

## Abstract

Iron is an essential nutrient for bacterial pathogenesis, but in the host, iron is tightly sequestered, limiting its availability for bacterial growth. Although this is an important arm of host immunity, most studies examine how bacteria respond to iron restriction in laboratory rather than host settings, where the microbiome can potentially alter pathogen strategies for acquiring iron. One of the most important transcriptional regulators controlling bacterial iron homeostasis is Fur. Here we used a combination of RNA-seq and chromatin immunoprecipitation (ChIP)-seq to characterize the iron-restricted and Fur regulons of the biofilm-forming opportunistic pathogen *Aggregatibacter actinomycetemcomitans*. We discovered that iron restriction and Fur regulate 4% and 3.5% of the genome, respectively. While most genes in these regulons were related to iron uptake and metabolism, we found that Fur also directly regulates the biofilm-dispersing enzyme Dispersin B, allowing *A*. *actinomycetemcomitans* to escape from iron-scarce environments. We then leveraged these datasets to assess the availability of iron to *A*. *actinomycetemcomitans* in its primary infection sites, abscesses and the oral cavity. We found that *A*. *actinomycetemcomitans* is not restricted for iron in a murine abscess mono-infection, but becomes restricted for iron upon co-infection with the oral commensal *Streptococcus gordonii*. Furthermore, in the transition from health to disease in human gum infection, *A*. *actinomycetemcomitans* also becomes restricted for iron. These results suggest that host iron availability is heterogeneous and dependent on the infecting bacterial community.

## Author Summary

One of the most well-studied phenomena in microbiology is nutritional immunity, or how the host withholds nutrients such as iron to combat infection. As part of this, researchers have characterized how many pathogens respond to iron restriction. However, these studies are often conducted in laboratory media rather than the host. As a result, they overlook how the host environment, such as its microbiome, might alter pathogen behavior regarding iron during infection. To address this gap, we used an opportunistic pathogen that causes abscess and oral cavity infections. We defined how it responds to iron restriction *in vitro* and then used this data to assess its iron status *in vivo*. Our results show that in mono-culture abscesses the pathogen is not starved for iron but in co-culture abscesses and multispecies gum disease it is starved for iron. Therefore, host environments are not uniformly restricted for iron, and the microbiome can modulate iron availability to pathogens.

## Introduction

In 1944, researchers Schade and Caroline found that by adding egg whites to cultures of various microorganisms, they could stunt microbial growth. Remarkably, they could only relieve this inhibition by supplementing the cultures with iron [1]. This was the first suggestion that iron, an essential nutrient for most microbes, is withheld by the host as a means to restrict infection. Since then, an entire field has emerged on the role of iron in host immunity [2–6].

Most bacteria require at least 0.3–4 μM iron to grow [2]. Pathogens therefore meet a significant hurdle when they infect the human body, where most iron is sequestered. Inside cells, iron is bound by proteins such as hemoglobin and ferritin, and outside cells, iron is bound by proteins such as transferrin and lactoferrin [7]. As a result, free ionic iron is present in human fluids at only 10^−12^ μM [3], over 11 orders of magnitude below that needed by bacteria. Thus, compromises to “nutritional immunity” can greatly increase vulnerability to infection. Demonstrating this, patients with hyperferremia (iron overload) are more prone to infection, and supplementation of mammals with iron has been shown to enhance the virulence of microbial pathogens [4].

In addition to host measures, acquiring iron is made challenging because under oxidizing conditions, most iron is ferric (in the 3+ oxidation state), which is insoluble and limited in bioavailability [8]. Bacteria can circumvent these barriers by either releasing siderophore molecules that bind ferric iron with high affinity and return it to the cell or by displaying surface receptors that bind iron directly [9]. These strategies, however, must also be tightly regulated because excess iron is toxic [10]. Many bacteria regulate iron acquisition with the Ferric Uptake Regulator (Fur), a transcriptional regulator that alters gene expression in response to intracellular iron levels [11]. In general, holo-Fur (Fur bound to Fe^2+^) represses gene expression, whereas apo-Fur (Fur not bound to Fe^2+^) de-represses gene expression. Besides iron transport, Fur can also control many other aspects of bacterial physiology and behavior, including toxin production, stress resistance, and biofilm formation [11].

While most nutritional immunity studies center on the interaction between host and pathogen, polymicrobial interactions between pathogens and the host microbiota can also affect the success of pathogens in acquiring iron, either through competition [12, 13] or cooperation [14–16]. One of the most common infectious diseases, periodontitis (gum disease), is also influenced by iron [17–19]. Periodontitis is caused by the multispecies biofilm community that inhabits the gingival crevice, the pocket between the gum and tooth [20]. This site is partially anaerobic [21] and bathed in a serum exudate known as crevicular fluid. Crevicular fluid contains many potential iron sources for microbial growth including transferrin, lactoferrin, hemoglobin, and inorganic iron [22]. In healthy serum, the average total iron concentration is ~1 mg/l, whereas in periodontal crevicular fluid it increases to ~5 mg/l [22]. Although this suggests that iron is more bioavailable in disease, one of the most highly upregulated microbial community functions in human periodontitis is iron transport [23], suggesting instead that iron is limited. The bioavailability of iron in periodontitis is therefore unclear and may depend on multiple factors.

An important member of oral microbial communities is the Gram-negative bacterium *Aggregatibacter actinomycetemcomitans*, an opportunistic pathogen associated with aggressive periodontitis. Notably, *A*. *actinomycetemcomitans* not only infects the oral cavity but can also spread throughout the body to cause abscesses [24]. In regards to iron sources, *A*. *actinomycetemcomitans* can use ferrous (Fe^2+^) iron, ferric (Fe^3+^) iron, and hemoglobin but not transferrin or lactoferrin [25–27]. Like most pathogens, the ability of *A*. *actinomycetemcomitans* to access one of these iron sources critically determines its virulence [28, 29]. Despite this, only a few iron acquisition systems in *A*. *actinomycetemcomitans* have been characterized. These systems directly bind and transport iron [25–27] since *A*. *actinomycetemcomitans* cannot make siderophores [25], and many of them are controlled by the *A*. *actinomycetemcomitans* Fur homolog. However, outside these systems only a few targets of Fur in *A*. *actinomycetemcomitans* are known [30].

Despite the clear importance of iron to bacterial infection, we know virtually nothing about how *A*. *actinomycetemcomitans* acquires it *in vivo*. How does *A*. *actinomycetemcomitans* surmount iron restriction at its different infection sites? How does *A*. *actinomycetemcomitans* interact with other bacteria regarding iron? Most importantly, how does *A*. *actinomycetemcomitans* respond to iron restriction *in vivo* to optimize its growth and virulence? With these questions in mind, our primary goal was to comprehensively characterize the *A*. *actinomycetemcomitans* iron-restricted and Fur regulons. We then leveraged these datasets to assess the *A*. *actinomycetemcomitans* iron response in both murine and human polymicrobial infections to determine the impact of microbial community composition and disease status on iron availability.

## Results/Discussion

### The *A*. *actinomycetemcomitans* iron-restricted regulon

To characterize how *A*. *actinomycetemcomitans* responds to iron restriction, we performed RNA-seq to compare its gene expression between rich media and iron-chelated media (see Tables 1–2 in S1 Dataset for study design). Since *A*. *actinomycetemcomitans* does not grow planktonically, we grew it as colony biofilms. We also used anaerobic growth conditions since soluble Fe^2+^ predominates and can be readily chelated with 2,2’-dipyridyl to mimic iron-restricted conditions. In total we discovered 93 genes, representing 4% of the genome, which are differentially expressed in response to iron restriction (Table 3 in S1 Dataset). Of these, over twice as many were upregulated than downregulated, and upregulated genes generally had larger fold changes than downregulated genes (S1A Fig).

**Fig 1:**
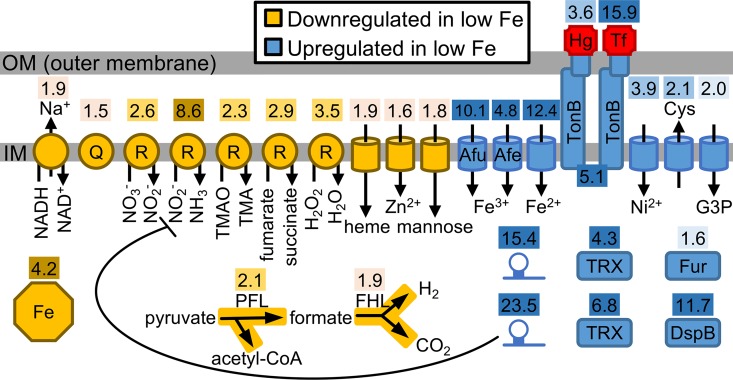
The *A*. *actinomycetemcomitans* iron-restricted regulon. Cellular processes differentially expressed by iron restriction. Shaded numbers above each process indicate fold change. 1.5–2.0 fold, light shade; >2.0–4.0 fold, medium shade; >4.0 fold, dark shade. Octagon, ferritin; Q, quinone; R, respiratory reductase; TMAO, trimethylamine N-oxide; TMA, trimethylamine; PFL, pyruvate formate lyase; FHL, formate hydrogen lyase; Afu and Afe, characterized transporters; Hg, hemoglobin; Tf, transferrin; Cys, cysteine; G3P, glycerol-3-phosphate; hairpin, sRNA; TRX, thioredoxin; DspB, Dispersin B.

Most genes downregulated in iron-restricted conditions encoded components of the anaerobic electron transport chain (Fig 1), suggesting that iron restriction hinders *A*. *actinomycetemcomitans* anaerobic respiration. These downregulated genes included the Na^+^-translocating NADH-quinone reductase [31], menaquinone biosynthesis [32], anaerobic respiratory reductases [33] as well as two major steps in pyruvate metabolism (Fig 1). The fact that many of these downregulated gene products contain iron suggests that restricting cellular iron usage is central to the *A*. *actinomycetemcomitans* iron starvation response. Supporting this, one of the most downregulated gene products in response to iron restriction was the iron storage protein ferritin (Fig 1).

Genes upregulated in response to iron restriction primarily encoded iron transporters and receptors. These include 5 inner membrane ABC transporters (including the characterized Afu [34] and Afe [35] systems), TonB [36], and 5 outer membrane TonB-dependent receptors (Fig 1). Substrates of these systems included inorganic iron, ferric iron siderophores, hemoglobin, and transferrin. Transporters for metals besides iron–nickel and zinc–were also differentially expressed (Fig 1). Although the significance of these non-iron transporters is unclear, co-expression of nickel with iron transporters has been observed in other bacteria [37].

Other processes upregulated in response to iron restriction included cysteine export by the CydDC transporter [38] (Fig 1). CydDC is required for the assembly of cytochrome *bd* (CydAB), and as CydAB is the sole aerobic respiratory oxidase in *A*. *actinomycetemcomitans* [39], this suggests that aerobic respiration in *A*. *actinomycetemcomitans* is stimulated by iron restriction. This is noteworthy considering that most of the *A*. *actinomycetemcomitans* anaerobic respiratory reductases were repressed by iron restriction. *A*. *actinomycetemcomitans* therefore seems to equate iron restriction with the presence of oxygen. Supporting this, a transporter for glycerol-3-phosphate, a carbon source that can only be catabolized by respiration [40], and 2 thioredoxins, implicated in resisting oxidative stress from oxygen [41], were also more highly expressed under iron restriction (Fig 1).

The only differentially expressed non-coding genes were 2 small RNAs (sRNA) upregulated by iron restriction (Fig 1). One of these sRNA was homologous to an iron-regulated sRNA in *H*. *influenzae* [42], and like *H*. *influenzae*, this sRNA in *A*. *actinomycetemcomitans* was also predicted to target asparagine biosynthesis (P = 0.03, TargetRNA2). The other sRNA was predicted to target nitrite reductase (P = 0.05, TargetRNA2 [43]), one of the most downregulated gene products in response to iron restriction.

### The *A*. *actinomycetemcomitans* Fur regulon

We next set out to characterize the *A*. *actinomycetemcomitans* Fur regulon with the goal of defining which iron-responsive genes were controlled by this transcriptional regulator. To do this, we used RNA-seq to compare gene expression of wild type *A*. *actinomycetemcomitans* to an isogenic Δ*fur* mutant (see Tables 1–2 in S1 Dataset for study design). Overall, we found that 386 genes, representing over 17% of the genome, were differentially expressed when Fur is absent, with slightly more genes activated than repressed (Table 3 in S1 Dataset). As expected, the Fur regulon extensively overlapped the iron-restricted regulon, with Fur-repressed genes mostly overlapping genes upregulated in iron-restricted conditions, and Fur-activated genes mostly overlapping genes downregulated in iron-restricted conditions (Table 4 in S1 Dataset). In total, the Fur regulon encompassed 70 of 93 genes in the iron-restricted regulon, indicating that as expected, Fur has a critical role in controlling *A*. *actinomycetemcomitans* iron homeostasis. Supporting this, all of the iron uptake systems that were upregulated in iron-restricted conditions were repressed by Fur, and all of the anaerobic respiratory reductases that were downregulated in iron-restricted conditions were activated by Fur (S1 Table).

Many Fur-activated genes were also related to carbon utilization (S1 Table). As these genes were not regulated by iron, we suspected that their control by Fur was due to indirect rather than direct regulatory changes. Supporting this, we identified 10 transcriptional regulators that were differentially expressed in the Δ*fur* mutant (S2A Fig). Furthermore, promoters of several Fur-activated genes contained a DNA binding motif for the cyclic AMP (cAMP) receptor protein (CRP) (S2B Fig), a regulator that alters gene expression in response to binding cAMP [44, 45]. As the CRP regulon in *A*. *actinomycetemcomitans* has been characterized [46], we could examine its overlap with the Fur regulon. This revealed that Fur-activated genes are enriched for both CRP-repressed (P = 1 x 10^−11^) and CRP-activated genes (P = 3 x 10^−4^, one-tailed Fisher’s exact test) (Table 4 in S1 Dataset). These results show that Fur expands its control over gene expression by acting indirectly through other transcriptional regulators including CRP.

**Fig 2:**
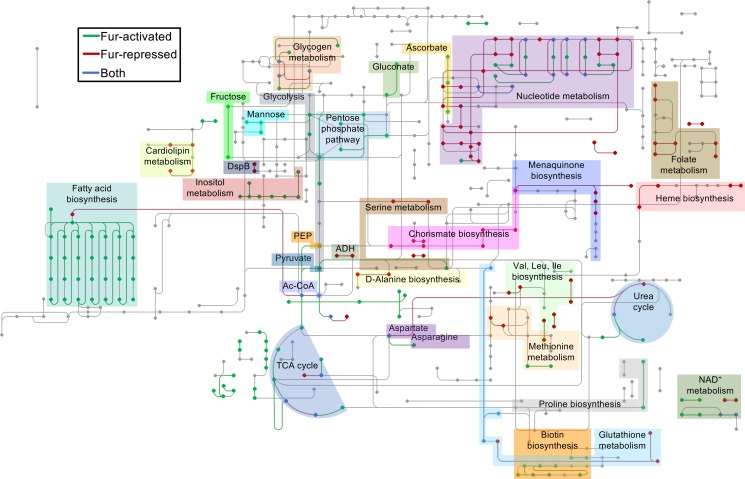
The *A*. *actinomycetemcomitans* Fur regulon. The metabolic network differentially expressed in the *Δfur* mutant. Legend: green, pathways downregulated in the *Δfur* mutant compared to the wild type (Fur-activated); red, pathways upregulated in the *Δfur* mutant compared to the wild type (Fur-repressed); blue, pathways mediated by both Fur-activated and -repressed genes. Dots and lines represent compounds and reactions, respectively. PEP, phosphoenolpyruvate; ADH, alcohol dehydrogenase; Ac-CoA, acetyl-CoA; TCA, tricarboxylic acid.

To gain a more in-depth understanding of the Fur regulon, we next used the KEGG resource [47] to reconstruct how Fur globally regulates *A*. *actinomycetemcomitans* metabolism (Fig 2, S1 Table). As Fur activity is essentially a proxy for cellular iron levels, this network could be interpreted as how *A*. *actinomycetemcomitans* metabolism might adapt to iron-restricted conditions. In general, we found that the Fur-activated regulon is metabolically diverse, encompassing several central metabolism and carbon utilization pathways. Central metabolism pathways activated by Fur included glycolysis, the pentose phosphate pathway, pyruvate metabolism, and the reductive TCA cycle, and carbon utilization pathways included those for ribose, ascorbate, citrate, gluconate, glycerol, and inositol.

In contrast, analogous pathways in the Fur-repressed regulon were much less diverse and suggest that iron-restricted *A*. *actinomycetemcomitans*, like other bacteria [48], primarily engages in fermentation, mediated by a D-lactate dehydrogenase and a zinc-dependent alcohol dehydrogenase. However, Fur both repressed and activated metabolic pathways related to amino acids, vitamins, and cofactors. For instance, chorismate (a precursor to aromatic amino acids) and serine biosynthesis were repressed by Fur, while tyrosine and serine transport were activated by Fur. Other processes regulated by Fur included autoinducer-2 (AI-2) signaling, toxin production, and biofilm formation. Specifically, the Lsr AI-2 transporter [49], leukotoxin [50], and tight adherence [51] pili that mediate surface attachment were activated by Fur, while cytolethal distending toxin [52] was repressed by Fur.

### Iron and Fur regulate Dispersin B

**Fig 3:**
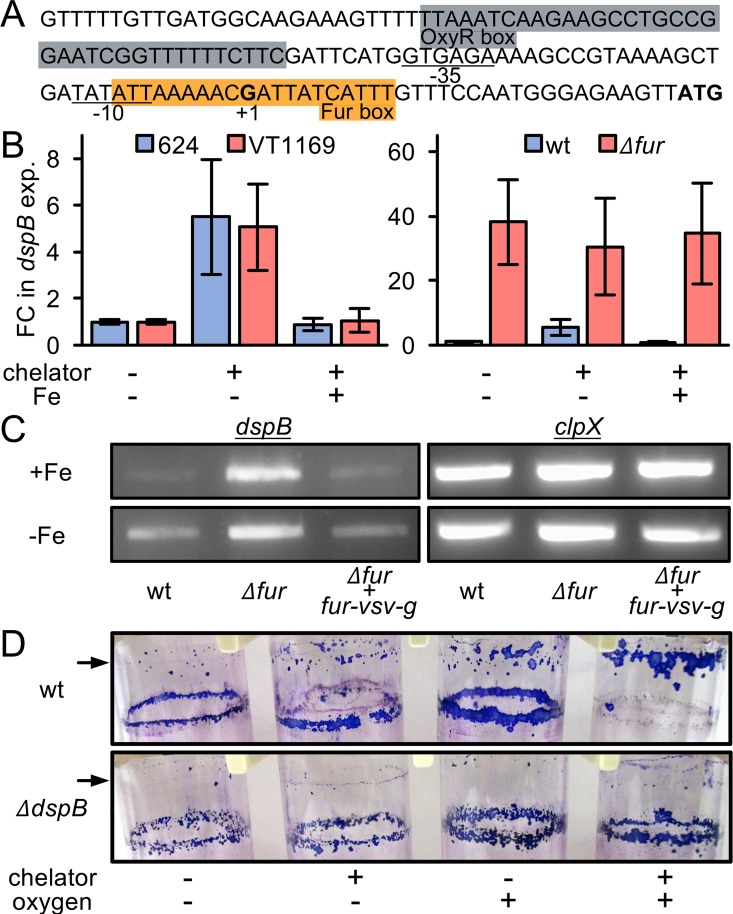
Iron and Fur regulate Dispersin B. (A) Structure of the *dspB* promoter. Gray, OxyR box; orange, Fur box; underlined, -35 and -10 regions; +1, transcriptional start site; bold, start codon. (B) *dspB* transcription in colony biofilms was measured using a *dspB* promoter-*lacZ* transcriptional fusion. Left panel: blue, *A*. *actinomycetemcomitans* strain 624; red, *A*. *actinomycetemcomitans* strain VT1169. Right panel: blue, *A*. *actinomycetemcomitans* strain 624 wild type (wt); red, *A*. *actinomycetemcomitans* strain 624 *Δfur* (*Δfur*). Chelator is 250 μM 2,2’-dipyridyl, and Fe is 250 μM FeSO_4_. Y axis is fold change (FC) in *dspB* expression relative to no chelator (-chelator) and no FeSO_4_ (-Fe) addition. Error bars represent standard deviation (n = 3). (C) *dspB* mRNA levels in colony biofilms was measured using reverse transcriptase PCR in iron-replete (+Fe) and iron-restricted (-Fe) conditions. *clpX* serves as a control that is not regulated by iron or Fur. Wild type (wt), *Δfur* (*Δfur*), *Δfur* + *fur-vsv-g* (*Δfur* genetically complemented with VSV-G tagged Fur). (D) Biofilm dispersal assay. A second, higher ring biofilm (indicated by arrow) indicates dispersal. The purple stain is crystal violet. Chelator is 250 μM 2,2’-dipyridyl; -oxygen is anaerobic growth; +oxygen is aerobic growth.

Iron restriction also caused Fur-mediated upregulation of the gene encoding Dispersin B (DspB) (Fig 1, S1 Table), an enzyme produced by *A*. *actinomycetemcomitans* to disperse from biofilms [53]. Previously we showed that *dspB* transcription is increased during aerobic growth via the transcriptional regulator OxyR [54]; however, the fact that we performed our experiments under strictly anaerobic conditions suggests that *dspB* transcription is also controlled by iron in an oxygen-independent manner. Thus, we hypothesized that iron restriction would induce transcription of *dspB* in a Fur-dependent manner and subsequently lead to dispersal of *A*. *actinomycetemcomitans* from biofilms. To test this hypothesis, we first searched the *dspB* promoter for a Fur binding motif. This revealed a sequence that overlaps the *dspB* transcriptional start site and lies downstream of the reported [54] OxyR binding motif (Fig 3A). We then used a *dspB* promoter-*lacZ* transcriptional fusion to measure how *dspB* transcription is impacted by iron restriction. First we grew *A*. *actinomycetemcomitans* as colony biofilms under anaerobic conditions, and then to restrict iron, we transferred these biofilms to media containing an iron chelator. As expected, *A*. *actinomycetemcomitans* induced transcription of *dspB* >5 fold upon iron restriction, and we observed this effect in two different strains of *A*. *actinomycetemcomitans* (Fig 3B), 624 (the primary strain used in this study) and VT1169. Furthermore, this effect was iron-specific as addition of FeSO_4_ to the chelated media abolished *dspB* induction (Fig 3B). We then tested the *Δfur* mutant under the same conditions and observed a >30 fold induction of *dspB* transcription. However, this induction occurred irrespective of iron levels (Fig 3B) confirming that Fur represses *dspB*. Genetic complementation of the *Δfur* mutant restored responsiveness to exogenous iron levels (Fig 3C) indicating that the response was specific to Fur.

We next used a previously described biofilm dispersal assay [54] to test if iron restriction triggers *A*. *actinomycetemcomitans* biofilm dispersal. This assay takes advantage of the fact that when *A*. *actinomycetemcomitans* is grown with shaking in a glass test tube, it forms a “ring biofilm” on the test tube (Fig 3D). The assay works by first forming a ring biofilm in a low volume of media and then adding more media. If a second biofilm then forms above the first biofilm, dispersal has occurred. Using this assay, we found that *A*. *actinomycetemcomitans* can be induced to disperse from biofilms by adding an iron chelator (Fig 3D). Notably, biofilms dispersed even under anaerobic conditions. Although the assay is qualitative, we could also observe that iron-restricted biofilms dispersed even further in the presence of oxygen (Fig 3D). This suggests that iron restriction and aerobic growth conditions can work synergistically to promote biofilm dispersal. Together, these results show that, in addition to oxygen and OxyR, *dspB* is regulated by iron and Fur, and this regulation mediates *A*. *actinomycetemcomitans* biofilm dispersal in response to iron restriction.

### The direct *A*. *actinomycetemcomitans* Fur regulon

One important drawback of our experimental approach for defining the Fur regulon is the inability to distinguish between direct and indirect gene regulation. We therefore used ChIP-seq to identify Fur binding sites (see Tables 1–2 in S1 Dataset for study design). To perform ChIP-seq, we complemented the *Δfur* mutant with a version of Fur tagged with the VSV-G epitope, allowing for immunoprecipitation. Importantly, VSV-G tagged Fur was expressed from its native promoter on a low-copy plasmid, and it genetically complemented the *Δfur* mutant (Fig 3C). In taking this approach, we hoped to prevent artefactual binding events that could arise from overexpression.

**Fig 4:**
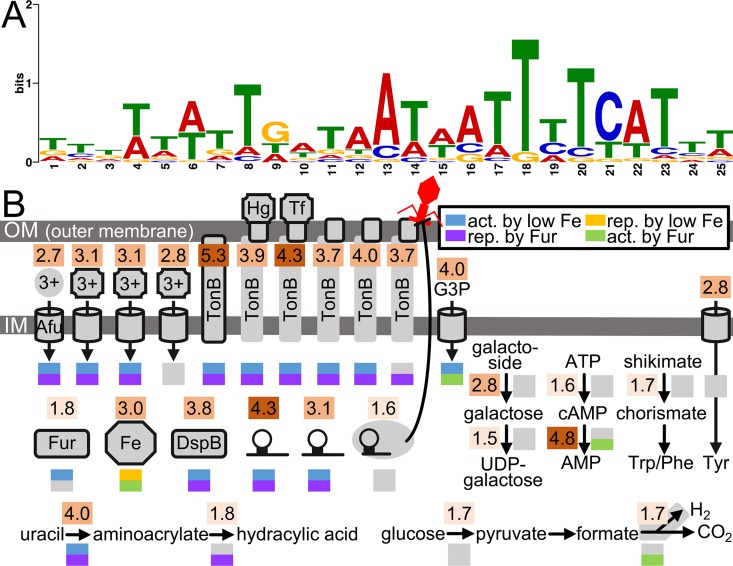
The *A*. *actinomycetemcomitans* direct Fur regulon. (A) Sequence logo representation of the Fur binding motif generated from all Fur-bound promoters (False Discovery Rate < 0.1) as outlined in Materials and Methods. The height of each base represents its frequency of occurrence. (B) Cellular processes directly regulated by Fur. Each process is encoded by a gene(s) whose promoter is bound by Fur. Shaded numbers above each process indicate fold enrichment of the ChIP to input DNA signal. 1.5–2.0 fold, light shade; >2.0–4.0 fold, medium shade; >4.0 fold, dark shade. Colored boxes below each process indicate transcriptional regulation by iron (blue or orange), Fur (purple or green), or neither (gray). Act. Is activated, and rep. is repressed. Afu, characterized transporter; 3+ in circle, free ferric iron; 3+ in square, ferric iron siderophore; Hg, hemoglobin; Tf, transferrin; octagon, ferritin; DspB, Dispersin B; hairpin, sRNA; hairpin in gray oval, CRISPR inhibiting a phage at the cell surface; G3P, glycerol-3-phosphate; cAMP, cyclic AMP; Trp, tryptophan; Phe, phenylalanine; Tyr, tyrosine.

In total, we identified 67 promoter regions, representing 77 genes that are directly bound by Fur in either iron-replete or iron-restricted conditions. After accounting for potential operons, this total increased to 91 genes, revealing that Fur directly regulates 3.5% of the genome (Table 3 in S1 Dataset). To our surprise, only 41 of the 91 Fur-bound promoters were differentially controlled in iron-restricted conditions or in the *Δfur* mutant (S1C Fig). As this has been seen in other bacteria [55], this phenomenon may be more widespread than expected. Although RNA-seq suggested Fur activates as much as it represses gene expression (Table 3 in S1 Dataset), ChIP-seq revealed that Fur primarily represses gene expression (Table 6 in S1 Dataset), with over 4 times as many genes being directly repressed by Fur than activated. As expected, promoters directly bound by Fur contained a sequence similar to reported [37, 55] Fur binding motifs (Fig 4A).

In regards to function, many of the genes directly regulated by Fur were related to iron homeostasis as well as other metabolic processes (Fig 4B). Iron transporters directly regulated by Fur included the Afu ferric iron ABC transporter [34], 3 ferric iron siderophore transporters, TonB, and 5 TonB-dependent receptors, including those for hemoglobin and transferrin [26]. Interestingly, one ferric iron siderophore transporter was not differentially expressed in either iron-restricted conditions or in the *Δfur* mutant (Fig 4B). Other iron-related proteins directly regulated by Fur included ferritin and Fur itself, demonstrating that as in many other bacteria with Fur homologs [11], Fur in *A*. *actinomycetemcomitans* is autoregulated.

Metabolic processes directly regulated by Fur included glycerol transport, galactoside degradation, aromatic amino acid metabolism, glucose catabolism, uracil degradation, and the biosynthesis of NAD^+^ and folate (Fig 4B). ChIP-seq also revealed that Fur controls both the biosynthesis and degradation of cAMP (Fig 4B), explaining the regulatory link we discovered between the Fur and CRP regulons (S2 Fig). Even though these two regulons were highly overlapping (Table 4 in S1 Dataset), CRP was not among the transcriptional regulators differentially expressed in the *Δfur* mutant (S2A Fig). This suggested control at the post-transcriptional level. As revealed by ChIP-seq, Fur exerts this control over CRP by regulating intracellular amounts of cAMP. Similar connections between Fur and CRP have been described in other bacteria [44, 45].

While most Fur-bound promoters were positioned ahead of coding genes, the direct Fur regulon also comprised 5 sRNA (Table 3 in S1 Dataset), including the 2 sRNA that we found are upregulated by iron restriction (Fig 4B). The promoters of 2 CRISPR-associated (*cas*) genes were also bound by Fur (Fig 4B). Though the promoter of the CRISPR itself was not bound by Fur, 2 other CRISPR in the genome were differentially expressed in the *Δfur* mutant (Table 3 in S1 Dataset). As there are a total of 3 CRISPR in our strain, the collective findings of our RNA- and ChIP-seq data suggest that Fur contributes to the regulation of all 3 CRISPR in the genome. One explanation for why *A*. *actinomycetemcomitans* evolved to regulate CRISPR with Fur is that surface-displayed iron receptors can serve as attachment sites for bacteriophages [56] (Fig 4B).

Finally, we found that Fur directly binds the *dspB* promoter, revealing that its upregulation in both iron-restricted conditions and the *Δfur* mutant (Fig 3) was due to direct de-repression by Fur. Interestingly, we found that Fur also binds to a gene in the tight adherence pili locus. As RNA-seq showed that this locus is activated by Fur, this suggests a model where iron controls the entire *A*. *actinomycetemcomitans* biofilm cycle: in the absence of iron, Fur promotes biofilm dispersal by de-repressing Dispersin B (Fig 3), whereas in the presence of iron, Fur promotes surface attachment by activating the tight adherence pili. Altogether, our ChIP-seq dataset extended our understanding of the Fur regulon, beyond that provided by RNA-seq, and gave insight into Fur’s complex role in not only iron transport but also cAMP biogenesis, viral defense, and biofilm formation.

### The *A*. *actinomycetemcomitans* iron and Fur regulons in abscess infections

**Fig 5:**
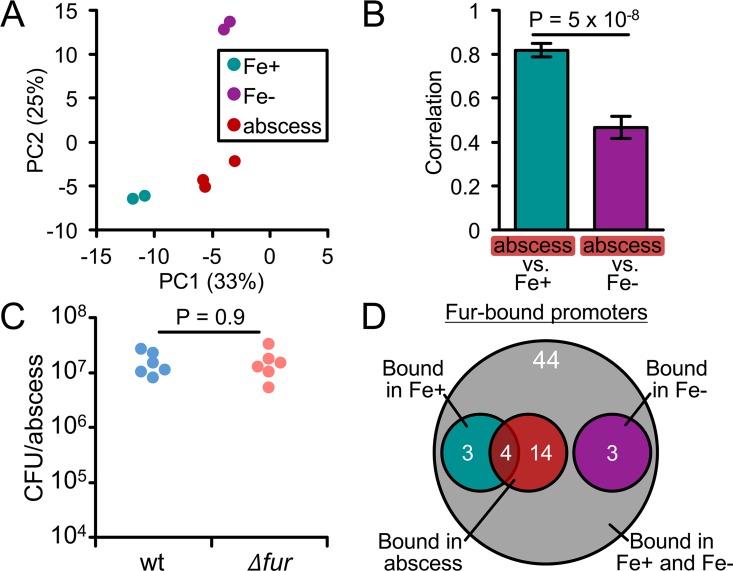
*A*. *actinomycetemcomitans* is not iron-restricted in murine abscess mono-infection. (A) Principal component analysis of the 93 genes regulated by iron. Each dot is a single replicate. Legend: Fe+, biofilm on rich media; Fe-, biofilm on iron-chelated media; abscess, wild-type abscess infection. Axes: Percentages are the amount of variation captured by each principal component. (B) Correlation analysis of the 93 genes regulated by iron. Spearman’s rank correlation was determined by comparing gene expression in wild-type *A*. *actinomycetemcomitans* abscess infection to Fe+ and Fe- *in vitro* biofilms. Error bars represent standard deviation (n = 6 pairwise comparisons). Significance was determined using a 2-tailed t test. (C) Survival of the wild type (wt) and *Δfur* mutant in abscesses. Each dot is a single abscess (n = 2 biological replicates). Significance was determined using a Mann-Whitney U test. Y axis represents colony forming units (CFU) per abscess after 3 days post-infection. (D) Venn diagram showing the overlap between the *in vitro* and *in vivo* ChIP-seq results.

After characterizing the iron and Fur regulons *in vitro*, we next set out to leverage these datasets for assessing iron availability in *A*. *actinomycetemcomitans* infections. While *A*. *actinomycetemcomitans* is most widely associated with oral cavity infections, it can also spread systemically and cause abscesses in many parts of the body [24]. Like the oral cavity, abscesses are thought to be strong targets of host iron restriction [57] suggesting that *A*. *actinomycetemcomitans* is likely restricted for iron in the abscess. To test this, we used principle component analysis (PCA) to determine whether *A*. *actinomycetemcomitans* gene expression in the abscess (using a published dataset [58]) is more similar to its gene expression in biofilms on rich (Fe+) media or on iron-chelated (Fe-) media. To our surprise, the PCA showed that the abscess lies closer to Fe+ than Fe- biofilms (Fig 5A). To quantify this relationship, we used Spearman’s correlation coefficient. This also showed that the abscess is more similar to Fe+ than Fe- biofilms (Fig 5B). We then repeated these analyses for genes in the Fur regulon, now comparing gene expression in the abscess to biofilms of the wild type and *Δfur* mutant. This showed that the abscess lies closer to and correlates better with the wild type than the *Δfur* mutant (S3 Fig). As gene expression in the *Δfur* mutant is essentially like that of iron-restricted wild type, the higher proximity of the abscess to the wild type suggests that gene expression within this infection better resembles that where iron is abundant than scarce. Together, these analyses indicate that *A*. *actinomycetemcomitans* is not restricted for iron in the abscess, contrasting our initial hypothesis.

To support this finding, we next decided to test the *Δfur* mutant in the abscess. In almost every pathogen and infection model tested, Fur is required for virulence [11]. Therefore, we reasoned that if iron is indeed not restricted in the abscess, Fur should not be required for *A*. *actinomycetemcomitans* virulence. As we anticipated, the *Δfur* mutant persisted just as well as the wild type in the abscess (Fig 5C), again indicating that *A*. *actinomycetemcomitans* does not face severe iron restriction in this host environment. As a final test, we also performed ChIP-seq on *A*. *actinomycetemcomitans* in the abscess, reasoning that we should be able to detect binding if iron is available. In total, we identified 18 promoters that are bound by Fur *in vivo* (Fig 5D). These promoters were a subset of the promoters that we identified are bound by Fur *in vitro*. Notably, the *in vivo*-bound promoters were enriched for promoters that are preferentially bound by Fur in the presence of iron (P = 0.08, one-tailed Fisher’s exact test) (Fig 5D, Table 5 in S1 Dataset), reaffirming our conclusion that *A*. *actinomycetemcomitans* is not iron-restricted in the abscess.

One possible explanation for this result is that *A*. *actinomycetemcomitans* elicits an immune response that fails to fully sequester iron from the abscess. Alternatively, iron could be low but spatially heterogeneous in the abscess (S5A Fig), forming iron-rich ‘patches’ that locally promote *A*. *actinomycetemcomitans* colonization and growth. Supporting this, *A*. *actinomycetemcomitans* proliferates in the abscess as small (<10 μm across) cell aggregates [54]. Future studies should therefore focus on examining the host response to *A*. *actinomycetemcomitans* using more direct means to quantitatively map iron levels, addressing the possibility that iron forms concentrated micron-scale patches and gradients in the abscess.

### The *A*. *actinomycetemcomitans* iron and Fur regulons in abscess co-infections

**Fig 6:**
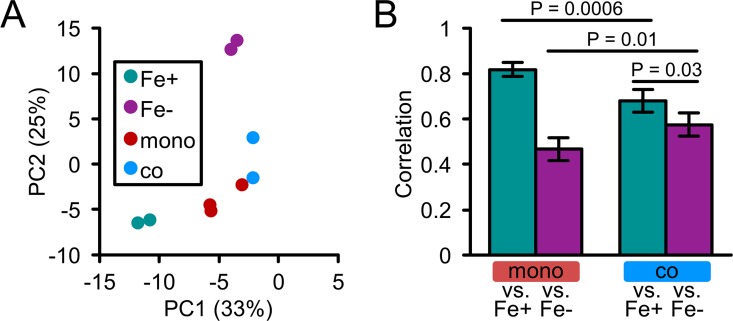
*A*. *actinomycetemcomitans* is iron-restricted in murine abscess co-infection. (A) Principal component analysis of the 93 genes regulated by iron. Each dot is a single replicate. Legend: Fe+, biofilm on rich media; Fe-, biofilm on iron-chelated media; mono, abscess mono-infection; co, abscess co-infection with *S*. *gordonii*. Axes: Percentages are the amount of variation captured by each principal component. (B) Correlation analysis of the 93 genes regulated by iron. Spearman’s rank correlation was determined by comparing Fe+ and Fe- *in vitro* biofilms to *A*. *actinomycetemcomitans* gene expression in mono-infection (mono vs. Fe+ and Fe-) or co-infection with *S*. *gordonii* (co vs. Fe+ and Fe-). Error bars represent standard deviation (n = 4–6 pairwise comparisons). Significance was determined using a 2-tailed t test.

As most pathogens cause infections as part of multispecies communities [59], we next sought to determine how co-infecting bacteria influence iron availability to *A*. *actinomycetemcomitans in vivo*. In the oral cavity, some of the most prevalent bacteria are Gram-positive streptococci [60]. Previously, we showed that intricate metabolic interactions, both cooperative [39] and competitive [54], with *Streptococcus gordonii* enhance *A*. *actinomycetemcomitans* virulence during abscess co-infection. Therefore, we conducted RNA-seq on abscesses co-infected with *A*. *actinomycetemcomitans* and *S*. *gordonii* (see Tables 1–2 in S1 Dataset for study design). Principal component analysis showed that co-infection, while positioned close to mono-infection, shifts the abscess towards Fe- biofilms (Fig 6A). Furthermore, the correlation between co-infection and Fe- biofilms was higher than that between mono-infection and Fe- biofilms (Fig 6B). In addition, genes upregulated in response to *S*. *gordonii* were enriched for genes upregulated by iron restriction (Table 7 in S1 Dataset) including 5 involved in iron uptake (S2 Dataset). Together, these results indicate that *A*. *actinomycetemcomitans* is restricted for iron during co-infection with *S*. *gordonii*.

How is iron restricted in co-infected abscesses? One possible mechanism for this interaction is direct interspecies competition for iron, as this has been reported in other mixed-species infections [18]. However, *S*. *gordonii* and other streptococci do not have an absolute growth requirement for iron [61], suggesting that interspecies competition is likely not responsible for reducing iron availability in co-infection. Supporting this, we found little evidence that *S*. *gordonii* differentially expresses genes related to iron homeostasis in either mono- or co-infection (Table 8 in S1 Dataset, S4 Dataset). Based on these data, we hypothesize that reduced iron in co-infection results from increased *A*. *actinomycetemcomitans* intraspecies competition (S5B Fig), a result of the 5–10 fold higher *A*. *actinomycetemcomitans* cell numbers observed during co-infection [39, 54, 62]. This higher bacterial burden could also enhance the host immune response and sequestration of iron within the abscess.

Since iron restriction induces *dspB* expression (Fig 1), a possible implication of the iron restriction associated with co-infection is that *A*. *actinomycetemcomitans* spatially reorganizes in response to *S*. *gordonii*. Previously we showed that *A*. *actinomycetemcomitans* proliferates in the abscess as small groups of cells (aggregates) and that the size of these aggregates is controlled by *dspB* [54]. Therefore, we anticipate that since *S*. *gordonii* restricts iron, *A*. *actinomycetemcomitans* forms smaller aggregates in co-infection than mono-infection. Of note, this spatial reorganization could facilitate iron acquisition since theoretically more cells per aggregate, due to the higher surface area to volume ratio, would have access to iron (S5C Fig). Current studies in our lab are aimed at addressing these possibilities.

### The *A*. *actinomycetemcomitans* iron and Fur regulons in human periodontitis

**Fig 7:**
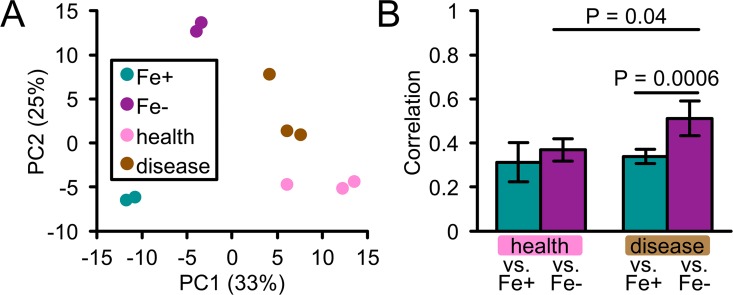
*A*. *actinomycetemcomitans* is iron-restricted in human periodontitis. (A) Principal component analysis of the 93 genes regulated by iron. Legend: Fe+, biofilm on rich media; Fe-, biofilm on iron-chelated media; health, *A*. *actinomycetemcomitans* from healthy human gingival crevice; disease, *A*. *actinomycetemcomitans* from diseased human gingival crevice. Axes: Percentages are the amount of variation captured by each principal component. (B) Correlation analysis of the 93 genes regulated by iron. Spearman’s rank correlation was determined by comparing Fe+ and Fe- *in vitro* biofilms to *A*. *actinomycetemcomitans* gene expression in healthy human gingival crevice samples (health vs. Fe+ and Fe-) or diseased human gingival crevice samples (disease vs. Fe+ and Fe-). Error bars represent standard deviation (n = 6 pairwise comparisons). Significance was determined using a 2-tailed t test.

Our abscess model provided a relatively simple *in vivo* environment for investigating the role of iron in interactions between *A*. *actinomycetemcomitans*, the host, and a co-infecting bacterium. To investigate the role of iron in a more complex polymicrobial environment, we next analyzed expression of the *A*. *actinomycetemcomitans* iron and Fur regulons during human periodontal disease. To do this, we extracted sequencing reads mapping to *A*. *actinomycetemcomitans* from a published meta-transcriptomics dataset comparing microbial gene expression from paired healthy and diseased (periodontitis) sites in the human oral cavity [63] (see Tables 1–2 in S1 Dataset for study design). Using principal component and correlation analyses, we showed that *A*. *actinomycetemcomitans* from healthy communities lies closer to Fe+ than Fe- biofilms (Fig 7A), but correlates to the same extent with both conditions (Fig 7B). In contrast, *A*. *actinomycetemcomitans* from diseased communities was closer to (Fig 7A) and correlated better with Fe- than Fe+ biofilms (Fig 7B). We obtained similar results for the Fur regulon (S3 Fig), specifically that disease was closer than health to the *Δfur* mutant. Furthermore, genes upregulated in periodontitis were enriched for both genes upregulated by iron restriction and repressed by Fur (Table 9 in S1 Dataset). These included several transporters for various iron sources. Notably, 3 of the 5 genes coding for the ferrous iron transporter were upregulated in periodontitis (S2 Dataset). This suggests that ferrous iron is an important iron source for *A*. *actinomycetemcomitans* in periodontal disease. We also found that the first gene in the *dspB* operon is upregulated in periodontitis. As DspB is stimulated not only by low iron (Fig 3) but also oxygen [54], we propose a model where *A*. *actinomycetemcomitans* disperses and migrates to deeper, anaerobic niches of the gingival pocket [21] during disease. As to why iron is restricted at diseased compared to healthy oral sites, we suspect that this phenomenon stems from a combination of the heightened host immune response (potentially resulting in greater iron sequestration) and the higher bacterial burden (potentially resulting in greater interspecies competition for iron) (S5D Fig).

### Conclusions

In summary, our *in vitro* analysis of the iron-restricted and Fur regulons of *A*. *actinomycetemcomitans* allowed us to gauge its behavior regarding iron levels in multiple infection sites. We discovered that Fur has a complex role, impacting not only *A*. *actinomycetemcomitans* iron homeostasis but also biofilm dispersal. Our observation that *A*. *actinomycetemcomitans* disperses from biofilms in iron-restricted environments suggests that this pathogen may overcome host iron restriction via actively promoting spatial re-localization. Of additional interest, we also found that the availability of iron to *A*. *actinomycetemcomitans* is heterogeneous *in vivo*. Indeed, while iron does not appear to be restricted in *A*. *actinomycetemcomitans* mono-species abscess infections, co-culture abscess infections and human gum disease appear to be iron-restricted infections. Collectively these results suggest that microbial pathogens use multiple methods (iron acquisition and spatial re-localization) to acquire iron during infection, and that co-infecting bacteria have a significant impact on whether a pathogen is restricted for iron.

## Materials and Methods

### Ethics statement

This study was performed in accordance with recommendations in the Guide for the Care and Use of Laboratory Animals of the National Institutes of Health. The animal protocol was approved by the Institutional Animal Care and Use Committee of The University of Texas at Austin (protocol number 00136). Mice were anesthetized with isoflurane delivered from a precision vaporizer and euthanized by asphyxiation with CO_2_ followed by cervical dislocation.

### Strains and growth conditions

*A*. *actinomycetemcomitans* 624 (a clinical isolate), *A*. *actinomycetemcomitans* VT1169 (a laboratory strain [64]) and *Streptococcus gordonii* Challis DL1.1 (ATCC 49818) were used in this study. Cultures were routinely grown in tryptic soy broth/agar (BD Difco) supplemented with 0.5% (w/vol) yeast extract (Fluka) (broth, TSBYE; agar, TSAYE) in a 5% CO_2_ atmosphere with shaking at 250 rpm for *A*. *actinomycetemcomitans*. Anaerobic cultures were grown in a vinyl chamber (Coy) supplied with the following gas mixture: 85% N_2_, 10% CO_2_, 5% H_2_. Colony biofilms were formed by spotting 100 μl of culture (adjusted to OD_600_ = 1) onto a polycarbonate, 0.2 μm pore size membrane (Whatman) placed onto the surface of a TSAYE plate.

### Strain construction

The *A*. *actinomycetemcomitans* 624 *Δfur* mutant was constructed by replacing the *fur* gene with a gene encoding spectinomycin resistance (*aad9*) by natural transformation as previously described [54] (see S2 Table for primer sequences). To construct the *A*. *actinomycetemcomitans* 624 strain for ChIP-seq, the *fur* gene and promoter region (~500 bp upstream of the *fur* start codon) were PCR-amplified from *A*. *actinomycetemcomitans* 624 genomic DNA with primers fur-pro-1-F and fur-tag-1-R, the reverse primer being designed to insert the VSV-G tag directly in front of the *fur* stop codon. This PCR product was then ligated into the TA vector pGEM-T Easy (Promega) and TSS transformed [65] into *E*. *coli* DH5α. A plasmid with the insert was then purified and used as the template for a second PCR with primers fur-pro-2-F and fur-tag-2-R, designed to incorporate KpnI restriction sites at the ends of the insert. Like the first PCR product, the second PCR product was cloned into pGEM-T Easy (Promega) and TSS transformed [65] into *E*. *coli* DH5α. A plasmid with the insert was then purified, digested with KpnI, and the VSV-G tagged *fur* gene was purified by gel extraction. The target vector pYGK [66] was also digested with KpnI, dephosphorylated with calf intestinal alkaline phosphatase (CIP), and purified. The KpnI-digested VSV-G tagged *fur* gene was then ligated into KpnI-digested, CIP-treated pYGK and TSS transformed into DH5α. This final plasmid was purified and electroporated as described [67] into the *A*. *actinomycetemcomitans* 624 *Δfur* mutant to generate the ChIP-seq strain. Plasmid inserts were sequenced at the UT Austin DNA Sequencing Facility.

### RNA-seq

Colony biofilms of the *A*. *actinomycetemcomitans* 624 wild type and *Δfur* mutant were prepared and grown overnight under anaerobic conditions. Biofilms were then transferred to fresh locations on the same plate, incubated for 2 hours, and then transferred to new plates, either TSAYE (+Fe condition) or TSAYE + 250 μM 2,2’-dipyridyl (-Fe condition). (This concentration of 2,2’-dipyridyl reduces the growth rate and yield of planktonic *A*. *actinomycetemcomitans* by approximately 50% [25], and fully restricts the growth of *A*. *actinomycetemcomitans* iron transport mutants on streak plates [27].) Biofilms were then further incubated for 2 hours, and following this they were stored in RNAlater solution. Per replicate, 8 colony biofilms were pooled together for each treatment (+Fe wild type, -Fe wild type, +Fe *Δfur*, -Fe *Δfur*), and altogether 2 replicates were performed, each on different days (Table 1 in S1 Dataset). Biofilm RNA was extracted with RNA-Bee (Tel-Test) according to the manufacturer’s protocol. Abscess RNA was extracted as previously described [58] from 1 or 2 pooled abscesses per biological replicate. Extracted RNA was treated with RQ1 DNase (Promega) to remove DNA contamination, and DNA removal was verified by PCR of the *clpX* gene. RNA integrity was verified by agarose gel separation of RNA denatured with NorthernMax-Gly loading dye (Ambion). Bacterial rRNA depletion, RNA fragmentation, and RNA-seq library preparation were performed as previously described [68], except that co-infected abscess RNA-seq libraries were rRNA-depleted with the MICROBEnrich and MICROBExpress kits (Ambion) and size-selected for fragments between 130–200 bp. Libraries were sequenced on 1x100 single-end Illumina HiSeq runs at the UT Austin Genome Sequencing and Analysis Facility. RNA-seq data were deposited into the NCBI Sequence Read Archive under accessions SRP081045 and SRP093165.

### ChIP-seq

Colony biofilms of *A*. *actinomycetemcomitans* 624 *Δfur* expressing VSV-G tagged *fur* were prepared and treated as described for the RNA-seq experiment, with the exception that growth plates included 10 μg/ml kanamycin to maintain the ChIP-seq plasmid. After 2 h incubation in the +Fe and -Fe conditions, biofilms were placed into 50 ml TSBYE + 1% formaldehyde per treatment, gently agitated for 20 min at room temperature, and then vigorously vortexed to dislodge the cells from the membranes. For abscess infections, the *A*. *actinomycetemcomitans* 624 *Δfur* strain expressing VSV-G tagged *fur* was first grown overnight in TSBYE + 10 μg/ml kanamycin under anaerobic conditions. Then, 3 ml of culture were washed and resuspended in 1 ml TSBYE, and 100 μl were injected into each thigh of three 10-week-old mice to form abscesses [62]. After 3 days, abscesses were harvested and each placed into 1 ml TSBYE + 5% formaldehyde. After overnight fixation, abscesses were homogenized, and the homogenates were collected by centrifugation and washed with TSBYE. Eight biofilms and 3 abscesses were used per experiment, and experiments were performed twice. At this point, the fixed biofilm cells and fixed abscess homogenates were subjected to the same ChIP procedure [37]. First, fixed samples were added with 0.5 M glycine to quench crosslinking. Samples were then washed with TBS (50 mM Tris-HCl, pH 7.5; 150 mM NaCl), each resuspended in 1 ml lysis buffer (10 mM Tris-HCl, pH 8; 100 mM NaCl; 1 mM EDTA; 0.5 mM EGTA; 0.1% deoxycholate; 0.5% N-lauroylsarcosine) + 1 mg/ml lysozyme + protease inhibitor (Sigma), and incubated at 37°C for 30 min. The samples were then chilled on ice, sonicated 2x for 5 s, or until the solution became clear, with a tip sonicator (QSonica) at 60% amplitude, and then further sonicated at 4°C for 20 min in 10 s on/10 s off cycles with a Q800R sonicator (QSonica) at 60% amplitude. Lysates were then separated from unlysed debris by centrifugation, and 25 μl of each clarified lysate was saved for the ChIP input control. A 1/10 volume of Triton X-100 (10% solution in lysis buffer) was then added to each sample, followed by 25 μl of Protein G Dynabeads (ThermoFisher) coated with anti-VSV-G monoclonal antibody (Sigma). The samples were rotated overnight at 4°C, and following this, each sample was washed 5x with 1 ml RIPA buffer (50 mM HEPES, pH 7.5; 500 mM LiCl; 1 mM EDTA; 1% Nonidet P-40; 0.7% deoxycholate), 1x with 1 ml TE (10 mM Tris-HCl, pH 8; 1 mM EDTA) + 50 mM NaCl, and resuspended in 100 μl EB (50 mM Tris-HCl, pH 7.5; 10 mM EDTA; 1% SDS). The samples were then incubated at 65°C for 30 min, separated from the Dynabeads by centrifugation, and further incubated overnight, along with the ChIP inputs, at 65°C to reverse crosslinks. Following this, the samples were brought up to 200 μl in volume with TE, incubated with 8 μl 10 mg/ml RNase A for 2 h at 37°C, and further incubated with 4 μl 20 mg/ml proteinase K for 2 h at 55°C. Finally, samples were purified with the ChIP DNA Clean & Concentrator kit (Zymo Research), and ChIP-seq libraries were prepared using the NEBNext ChIP-Seq Library Master Mix Set according to the manufacturer’s instructions. Libraries were sequenced on 1x75 single-end Illumina NextSeq runs at the UT Austin Genome Sequencing and Analysis Facility. ChIP-seq data were deposited into the NCBI Sequence Read Archive under accession SRP081045.

### Genome sequencing

*A*. *actinomycetemcomitans* 624 genome sequencing and assembly were performed as previously described [62]. Annotation was performed with PGAP [69], RAST [70], and KAAS [71]. Noncoding RNA sequences [58] were extracted from the D7S-1 genome with the bedtools getfasta function [72] and mapped to the *A*. *actinomycetemcomitans* 624 genome with bowtie2 v2.2.5 in very-sensitive-local mode [73]. Raw genome sequences were deposited into the NCBI Sequence Read Archive under accession SRP064936. The genome assembly was deposited into GenBank under accession CP012959.

### RNA-seq analysis

Raw reads were processed with cutadapt v1.9.1 (and higher) (http://cutadapt.readthedocs.org/en/stable/index.html) to (1) trim 3’ low-quality bases (cutoff: 15), (2) trim 3’ adaptors (sequence: AGATCGGAAGAGCACACGTCTGAACTCCAGTCAC), and (3) discard short reads (minimum length: 10 bases for non-human samples, or 15 bases for human samples,). Processed reads were mapped to the *A*. *actinomycetemcomitans* 624 or *S*. *gordonii* genome with bowtie2 v2.2.5 in very-sensitive-local mode [73], and only reads with mapping quality (MAPQ) scores ≥39 for non-human samples, or ≥20 for human samples, were kept for further analysis. Reads were counted per gene strand-specifically with the featureCounts function in Rsubread v1.20 (and higher) [74]. Raw read counts, excluding rRNA and tRNA, were adjusted for between-sample differences in sequencing depth with the estimateSizeFactors function in DESeq2 v1.10.1 (and higher) [75]. Raw read counts were also normalized for between-gene differences in GC content by including a normalization matrix. This normalization matrix was generated with the withinLaneNormalization function in EDASeq v2.4.1 (and higher) [76], and transformed to be on the scale of read counts, as described in the DESeq2 vignette. GC content values for the matrix were calculated with the nucBed function in bedtools v2.20 (and higher) [72]. Default DESeq2 parameters were used for estimating dispersions and performing the Wald test for differential expression analysis, and a multi-factor design, as described in the DESeq2 vignette, was used for paired analysis of healthy and diseased human from each patient [63]. Significance cutoffs were as follows. Iron-restricted, Fur, and co-infection regulons: log_2_ fold change (FC) > 0.5; adjusted P value < 0.05. Periodontitis: log_2_ FC > 0.5; non-adjusted P < 0.05. See Table 10 in S1 Dataset for a summary of the RNA-seq data analysis. Analyses were performed both locally and on the UT Austin Texas Advanced Computer Center.

### ChIP-seq analysis

Raw reads were processed as described for the RNA-seq analysis. Binding regions (peaks) were called with MOSAiCS v2.9.9 [77]. Adjustable MOSAiCS parameters were: fragLen, 200; binSize, 50; capping, 3; bgEst, automatic; FDR, 0.1. The control for colony biofilm samples was the *in vitro* ChIP input, and the control for abscess samples was the *in vivo* ChIP input. Promoters were defined for coding genes as the 200 and 50 bp up- and downstream of the start codon and for noncoding genes as the 100 and 25 bp up- and downstream of the start codon. The number of peaks overlapping each promoter was counted for each sample with the bedtools intersectBed function [72]. Peaks were only counted if they overlapped at minimum 50 bp of coding promoters or 25 bp of noncoding promoters. After tallying overlapping peaks, promoters were only considered bound by Fur if they overlapped a peak in at least 2 *in vitro* biofilm replicates. If one of these promoters also overlapped a peak in at least 1 of the abscess replicates, it was considered also bound by Fur *in vivo*. Promoters overlapping peaks in both Fe+ biofilm replicates were considered preferentially bound in the presence of iron, and promoters overlapping peaks in both Fe- biofilm replicates were considered preferentially bound in the absence of iron. See Tables 11–12 in S1 Dataset for a summary of the ChIP-seq data analysis. Analyses were performed both locally and on the UT Austin Texas Advanced Computer Center.

### Binding motif analysis

Promoter sequences were extracted from the *A*. *actinomycetemcomitans* 624 genome with the bedtools getfasta function [72] and submitted to MEME [78] to identify consensus binding motifs. MEME settings were: site distribution, zero or one occurrence per sequence; background model, 0-order model of sequences; minimum motif width, 15; maximum motif width, 25. A CRP box was identified among the promoters of genes activated by Fur, and a Fur box was identified among the promoters directly bound by Fur. Each binding motif was submitted to the MEME Suite program FIMO [78] to calculate the significance (FDR) of its occurrence within each promoter of its respective promoter set.

### sRNA target prediction

sRNA sequences were extracted from the *A*. *actinomycetemcomitans* 624 genome with the bedtools getfasta function [72] and submitted to TargetRNA2 [43] with default parameters and the *A*. *actinomycetemcomitans* strain D7S-1 genome selected. Predicted target sequences in the D7S-1 genome were mapped in fasta format to the *A*. *actinomycetemcomitans* 624 genome with bowtie2 v2.2.5 in very-sensitive-local mode [73].

### CRP regulon

The *A*. *actinomycetemcomitans* CRP regulon was determined with a microarray designed for strain HK1651 [46]. To use this data, *A*. *actinomycetemcomitans* 624 gene sequences in fasta format were mapped to the *A*. *actinomycetemcomitans* HK1651 genome with bowtie2 v2.2.5 in very-sensitive-local mode [73].

### Principal component analysis

Normalized read counts were transformed with the DESeq2 [75] rlog method with blindness set to false, as described in the DESeq2 vignette, and the PCA was performed with the prcomp function in R on the 93 genes in the iron-restricted regulon or the 218 genes in the Fur regulon.

### β-galactosidase assay

β-galactosidase assays were performed as previously described [54] using a chemiluminescent assay system (Galacto-Light Plus).

### Biofilm dispersal assay

The biofilm dispersal assay was performed as previously described [54].

### Abscess model

The murine abscess infection model was performed as previously described [39, 62], except that in the experiment testing the virulence of the *Δfur* mutant, anaerobic conditions were used for preparing the inoculum and plating serial dilutions of the abscess homogenates.

### Accession numbers

All RNA-seq and ChIP-seq files are available from the National Center for Biotechnology Information Sequence Read Archive (accession numbers SRP081045 and SRP093165).

## Supporting Information

S1 Fig Scatter plots of the *A*. *actinomycetemcomitans* iron-restricted and Fur regulons. (A) Genes differentially expressed in response to iron restriction. Y axis: fold change (FC) comparing iron-chelated to rich media. Legend: blue dots represent genes differentially expressed >4 fold comparing iron-chelated to rich media. (B) Genes differentially expressed in the *Δfur* mutant. Y axis: Fold change (FC) comparing the *Δfur* mutant to the wild type (wt). Left: comparison on rich media. Right: comparison on iron-chelated media. Legend: red dots represent genes that are regulated by both Fur and iron. (C) Genes whose promoters are bound by Fur. Rows: ChIP, all Fur-bound promoters; low Fe, Fur-bound promoters of genes differentially expressed in response to iron restriction; *Δfur*, Fur-bound promoters of genes differentially expressed in the *Δfur* mutant.
(TIF)

S2 Fig Indirect regulation in *A*. *actinomycetemcomitans Δfur*. (A) Transcriptional regulators differentially expressed in the *Δfur* mutant. The cellular process controlled by each regulator is indicated in parentheses. Colors: purple, repressed by Fur; green, activated by Fur. (B) A CRP binding motif was found with False Discovery Rate < 0.1 in 64 of the 218 promoters of Fur activated genes.
(TIF)

S3 Fig The *A*. *actinomycetemcomitans* Fur regulon *in vivo*. (A) Principal component analysis of the 218 genes differentially expressed in the *Δfur* mutant. Each dot is a single replicate. Legend: wt, wild-type biofilm on rich media; *Δfur*, *Δfur* biofilm on rich media; mono, abscess mono-infection; co, abscess co-infection with *S*. *gordonii*; health, *A*. *actinomycetemcomitans* from healthy human gingival crevice; disease, *A*. *actinomycetemcomitans* from diseased human gingival crevice. Axes: Percentages are the amount of variation captured by each principal component. (B) Correlation analysis of the 218 genes differentially expressed in the *Δfur* mutant. Spearman’s rank correlation was determined by comparing Fe+ and Fe- *in vitro* biofilms to *A*. *actinomycetemcomitans* gene expression in mono-infection (mono vs. Fe+ and Fe-), co-infection with *S*. *gordonii* (co vs. Fe+ and Fe-), healthy human gingival crevice samples (health vs. Fe+ and Fe-), or diseased human gingival crevice samples (disease vs. Fe+ and Fe-). Error bars represent standard deviation (n = 4–6 pairwise comparisons). Significance was determined using a 2-tailed t test (^a^, unpaired test; ^b^, paired test).
(TIF)

S4 Fig Cellular processes differentially expressed in periodontitis. Each shown process is encoded by a gene(s) that is regulated by iron restriction and/or Fur. Q, quinone; R, respiratory reductase; AI-2, autoinducer-2; GAP, glyceraldehyde-3P; Ac-CoA, acetyl-CoA; UMP, uridine monophosphate; 2+ in circle, free ferrous iron; 3+ in circle, free ferric iron; 3+ in square, ferric iron siderophore; Ala, alanine; Gly, glycine; hairpin, sRNA.
(TIF)

S5 Fig Model for iron dynamics in *A*. *actinomycetemcomitans* abscess and oral cavity infections. (A) Iron in mono-infected abscesses may be high (left) (e.g. if the host were not to fully sequester iron), low (middle), or low but spatially uneven (right), forming concentrated patches. These patches could induce *A*. *actinomycetemcomitans* to grow as aggregates. (B) *A*. *actinomycetemcomitans* in mono-infected abscesses (*Aa*, left) is not restricted for iron, but in co-infected abscesses with *S*. *gordonii* (*Aa* + *Sg*, right), higher *A*. *actinomycetemcomitans* titers may result in greater competition over iron between cells of *A*. *actinomycetemcomitans*. (C) In mono-infection (*Aa*, left), cells at the center of aggregates may not be restricted for iron, but in co-infection prior to dispersal (*Aa* + *Sg* pre-disp, middle), reduced iron may prevent these cells’ access to iron. Therefore, the formation of smaller aggregates after dispersal (*Aa* + *Sg* post-disp) may restore cellular access to iron. (D) The host immune response and higher bacterial burden associated with periodontitis (right) may create a larger iron gradient than seen in health (left), inducing *A*. *actinomycetemcomitans* to spread deeper into the gingival crevice (space between tooth and gum).
(TIF)

S1 Dataset (DOCX)

S2 Dataset (XLSX)

S3 Dataset (XLSX)

S4 Dataset (XLSX)

S1 Table (DOCX)

S2 Table (DOCX)
