# Supplementary figures and images for: Microbial Community Composition Impacts Pathogen Iron Availability during Polymicrobial Infection
Source: PLoS Pathog. 2016 Dec 14;12(12):e1006084. doi: 10.1371/journal.ppat.1006084 (PMC5156373; doi:10.1371/journal.ppat.1006084)

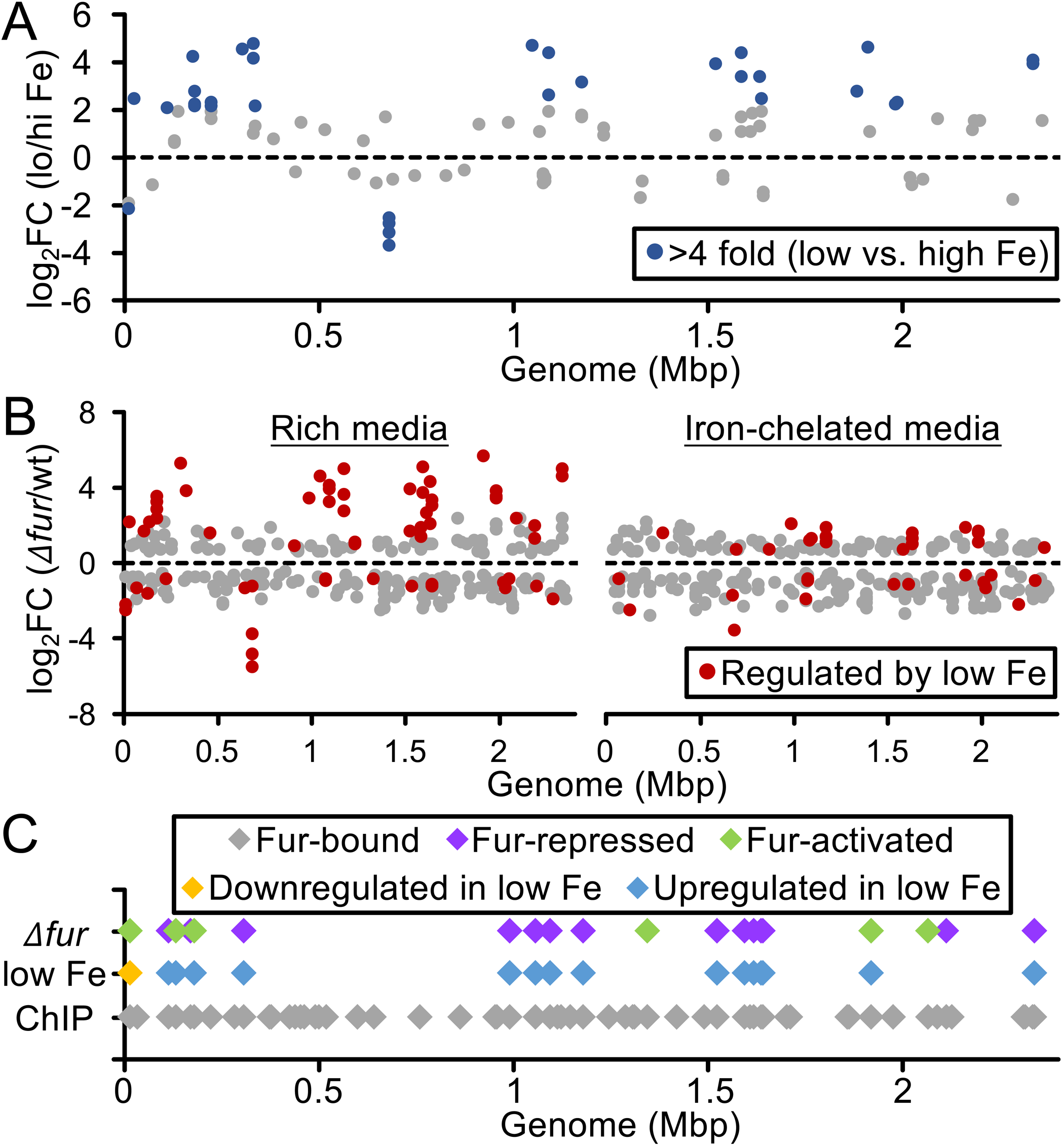

Supplement: S1 Fig — (A) Genes differentially expressed in response to iron restriction. Y axis: fold change (FC) comparing iron-chelated to rich media. Legend: blue dots represent genes differentially expressed >4 fold comparing iron-chelated to rich media. (B) Genes differentially expressed in the Δfur mutant. Y axis: Fold change (FC) comparing the Δfur mutant to the wild type (wt). Left: comparison on rich media. Right: comparison on iron-chelated media. Legend: red dots represent genes that are regulated by both Fur and iron. (C) Genes whose promoters are bound by Fur. Rows: ChIP, all Fur-bound promoters; low Fe, Fur-bound promoters of genes differentially expressed in response to iron restriction; Δfur, Fur-bound promoters of genes differentially expressed in the Δfur mutant. (TIF) [file ppat.1006084.s001.tif]

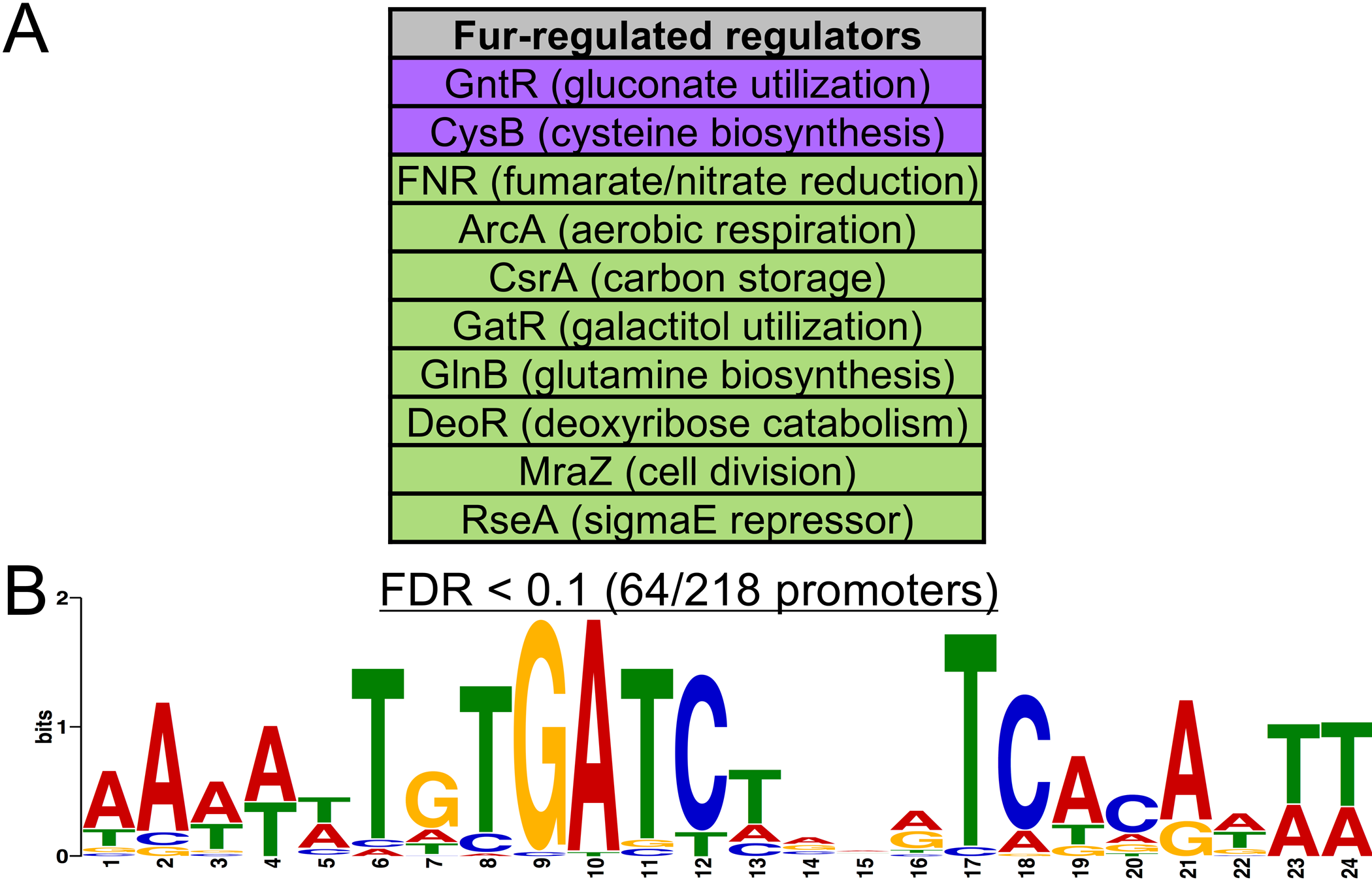

Supplement: S2 Fig — (A) Transcriptional regulators differentially expressed in the Δfur mutant. The cellular process controlled by each regulator is indicated in parentheses. Colors: purple, repressed by Fur; green, activated by Fur. (B) A CRP binding motif was found with False Discovery Rate < 0.1 in 64 of the 218 promoters of Fur activated genes. (TIF) [file ppat.1006084.s002.tif]

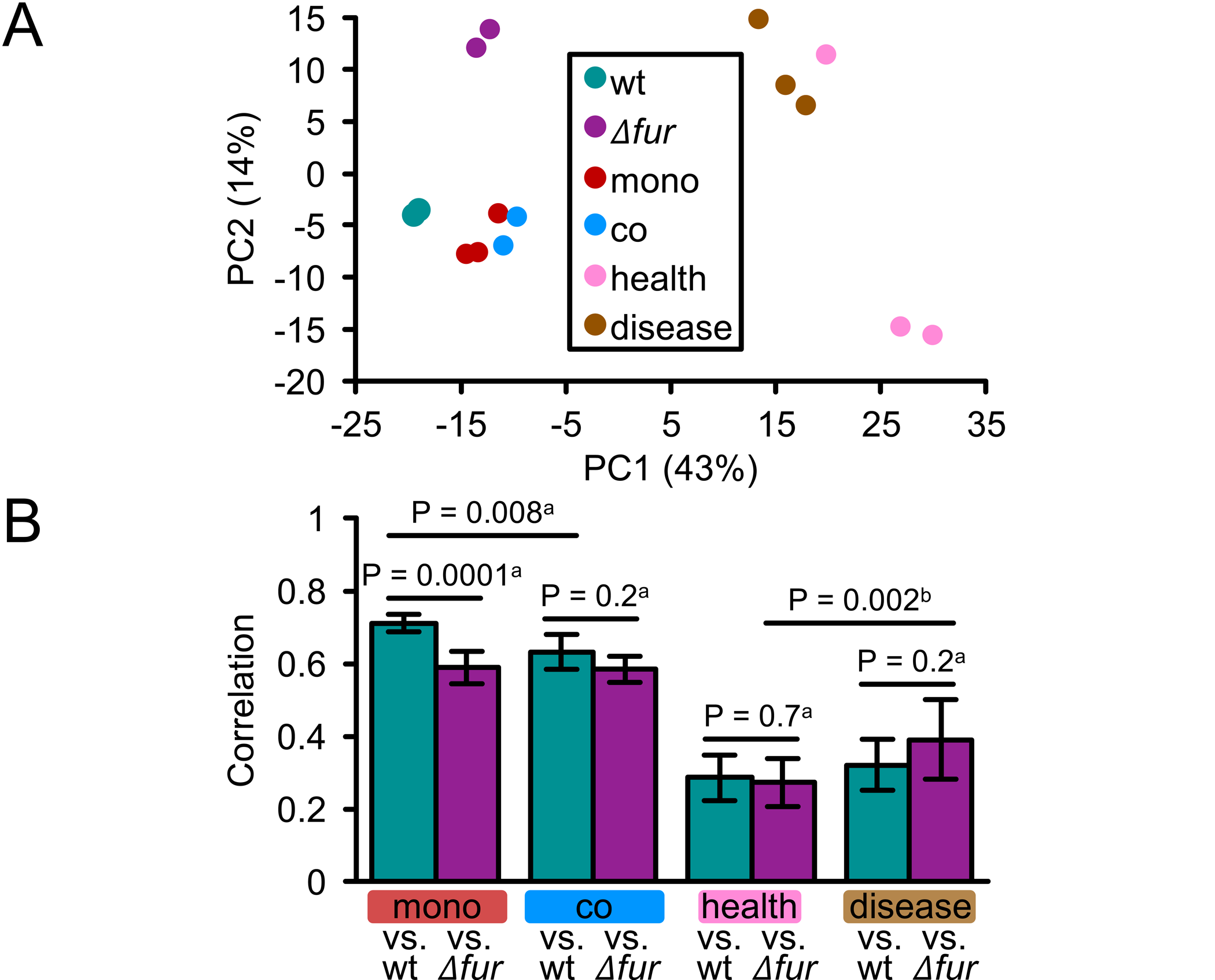

Supplement: S3 Fig — (A) Principal component analysis of the 218 genes differentially expressed in the Δfur mutant. Each dot is a single replicate. Legend: wt, wild-type biofilm on rich media; Δfur, Δfur biofilm on rich media; mono, abscess mono-infection; co, abscess co-infection with S. gordonii; health, A. actinomycetemcomitans from healthy human gingival crevice; disease, A. actinomycetemcomitans from diseased human gingival crevice. Axes: Percentages are the amount of variation captured by each principal component. (B) Correlation analysis of the 218 genes differentially expressed in the Δfur mutant. Spearman’s rank correlation was determined by comparing Fe+ and Fe- in vitro biofilms to A. actinomycetemcomitans gene expression in mono-infection (mono vs. Fe+ and Fe-), co-infection with S. gordonii (co vs. Fe+ and Fe-), healthy human gingival crevice samples (health vs. Fe+ and Fe-), or diseased human gingival crevice samples (disease vs. Fe+ and Fe-). Error bars represent standard deviation (n = 4–6 pairwise comparisons). Significance was determined using a 2-tailed t test (a, unpaired test; b, paired test). (TIF) [file ppat.1006084.s003.tif]

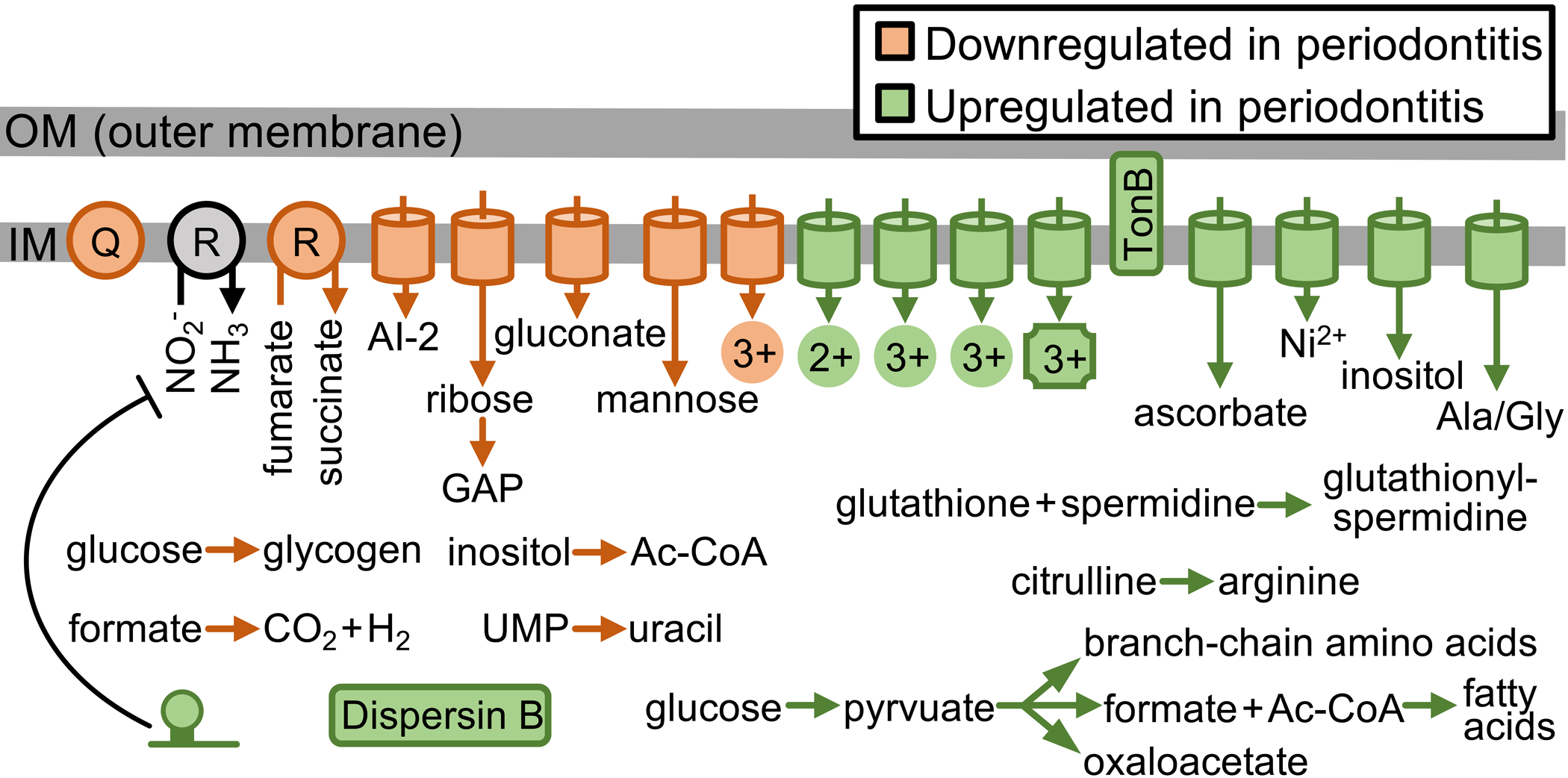

Supplement: S4 Fig — Each shown process is encoded by a gene(s) that is regulated by iron restriction and/or Fur. Q, quinone; R, respiratory reductase; AI-2, autoinducer-2; GAP, glyceraldehyde-3P; Ac-CoA, acetyl-CoA; UMP, uridine monophosphate; 2+ in circle, free ferrous iron; 3+ in circle, free ferric iron; 3+ in square, ferric iron siderophore; Ala, alanine; Gly, glycine; hairpin, sRNA. (TIF) [file ppat.1006084.s004.tif]

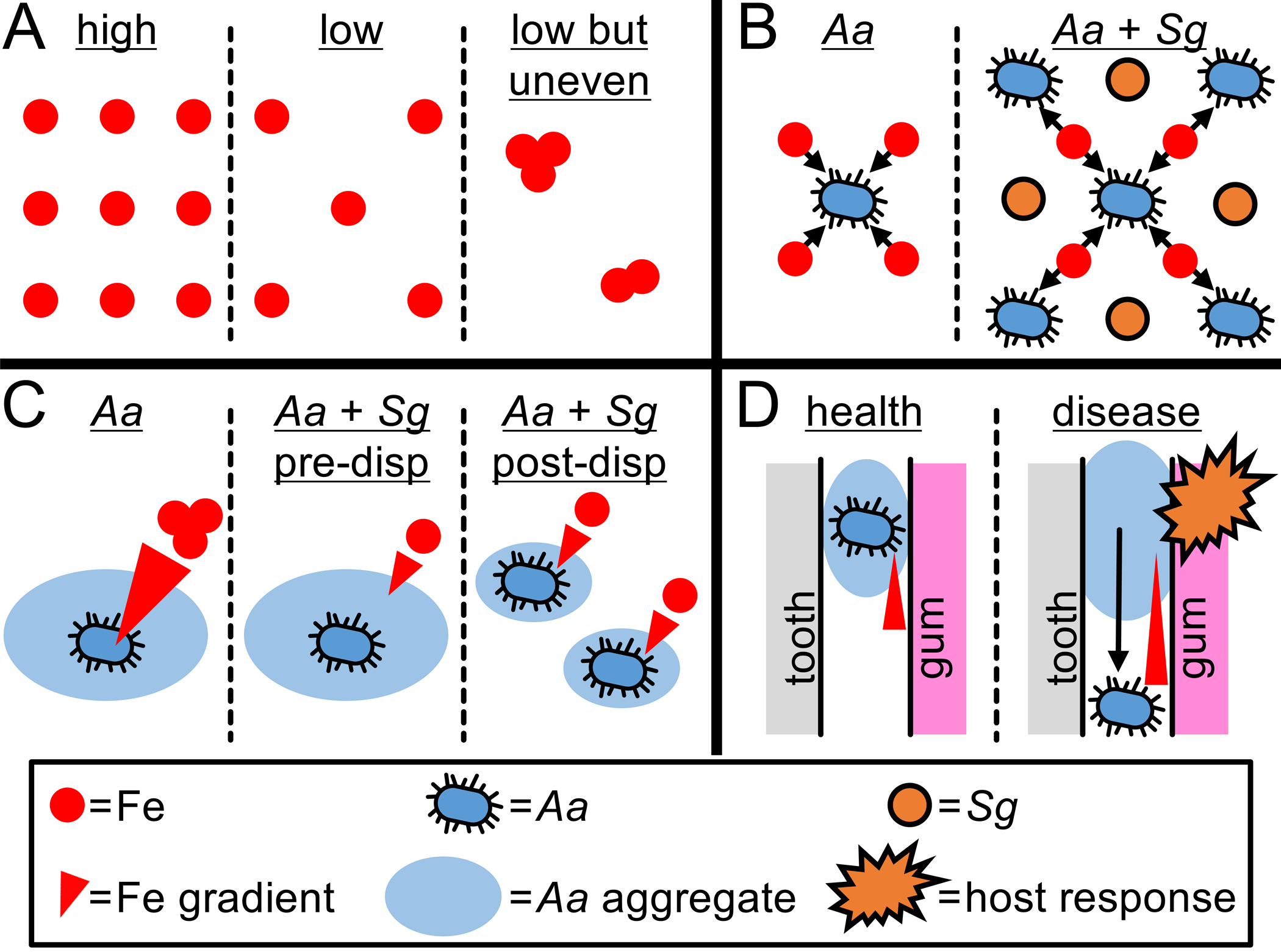

Supplement: S5 Fig — (A) Iron in mono-infected abscesses may be high (left) (e.g. if the host were not to fully sequester iron), low (middle), or low but spatially uneven (right), forming concentrated patches. These patches could induce A. actinomycetemcomitans to grow as aggregates. (B) A. actinomycetemcomitans in mono-infected abscesses (Aa, left) is not restricted for iron, but in co-infected abscesses with S. gordonii (Aa + Sg, right), higher A. actinomycetemcomitans titers may result in greater competition over iron between cells of A. actinomycetemcomitans. (C) In mono-infection (Aa, left), cells at the center of aggregates may not be restricted for iron, but in co-infection prior to dispersal (Aa + Sg pre-disp, middle), reduced iron may prevent these cells’ access to iron. Therefore, the formation of smaller aggregates after dispersal (Aa + Sg post-disp) may restore cellular access to iron. (D) The host immune response and higher bacterial burden associated with periodontitis (right) may create a larger iron gradient than seen in health (left), inducing A. actinomycetemcomitans to spread deeper into the gingival crevice (space between tooth and gum). (TIF) [file ppat.1006084.s005.tif]
