# Supplementary material for: Microbial Community Composition Impacts Pathogen Iron Availability during Polymicrobial Infection
Source: PLoS Pathog. 2016 Dec 14;12(12):e1006084. doi: 10.1371/journal.ppat.1006084 (PMC5156373; doi:10.1371/journal.ppat.1006084)
Supplement: S1 Dataset — (DOCX) [file ppat.1006084.s006.docx]

**Table 1. Summary of study design.**

| Data generated in this study. | | | | | | | | |
| --- | --- | --- | --- | --- | --- | --- | --- | --- |
| Experiment | | | Replicate 1 | | | Replicate 2 | | |
| *in vitro* | RNA-seq | *Aa* wt | +Fe | 8 biofilms | | +Fe | | 8 biofilms |
|  |  |  | -Fe | 8 biofilms | | -Fe | | 8 biofilms |
|  |  | *Aa 𝛥fur* | +Fe | 8 biofilms | | +Fe | | 8 biofilms |
|  |  |  | -Fe | 8 biofilms | | -Fe | | 8 biofilms |
|  | ChIP-seq | *Aa 𝛥fur*  +  *fur-vsv-g* | +Fe | 8 biofilms | | +Fe | | 8 biofilms |
|  |  |  | -Fe | 8 biofilms | | -Fe | | 8 biofilms |
| *in vivo* | RNA-seq | *Sg* | 1 murine abscesses | | | 1 murine abscesses | | |
|  |  | *Aa* + *Sg* | 2 murine abscesses | | | 2 murine abscesses | | |
| Data generated in Jorth *et al.*, 2013 | | | | | | | | |
| Experiment | | | Replicate 1 | | Replicate 2 | | Replicate 3 | |
| *in vivo* | RNA-seq | *Aa* | 6 abscesses | | 2 abscesses | | 2 abscesses | |
| Data generated in Jorth *et al.*, 2014 | | | | | | | | |
| Experiment | | | Patient 1 | | Patient 2 | | Patient 3 | |
| *in vivo* | meta-transcriptomics | | healthy human  dental plaque | | healthy human  dental plaque | | healthy human  dental plaque | |
|  |  |  | diseased human dental plaque | | diseased human dental plaque | | diseased human dental plaque | |

**Table 2. Comparisons made between datasets in each figure.**

| Figure | Data type | Comparison |
| --- | --- | --- |
| 1, S1A | Differential gene expression | *Aa* wt -Fe biofilms vs. *Aa* wt +Fe biofilms |
| 2, S1B | Differential gene expression | *Aa* *𝛥fur* +Fe biofilms vs. *Aa* wt +Fe biofilms  *Aa* *𝛥fur* -Fe biofilms vs. *Aa* wt -Fe biofilms |
| 4B, S1C | DNA binding | ChIP on *Aa* *𝛥fur* + *fur-vsv-g* +Fe biofilms vs. DNA input  ChIP on *Aa* *𝛥fur* + *fur-vsv-g* -Fe biofilms vs. DNA input |
| 5A-B | Normalized expression | *Aa* wt +Fe biofilms  *Aa* wt -Fe biofilms  *Aa* mono-abscess |
| 5D | DNA binding | ChIP on *Aa* *𝛥fur* + *fur-vsv-g* +Fe biofilms vs. DNA input  ChIP on *Aa* *𝛥fur* + *fur-vsv-g* -Fe biofilms vs. DNA input  ChIP on *Aa* *𝛥fur* + *fur-vsv-g* mono-abscess vs. DNA input |
| 6 | Normalized expression | *Aa* wt +Fe biofilms  *Aa* wt -Fe biofilms  *Aa* mono-abscess  *Aa* + *Sg* co-abscess |
| 7 | Normalized expression | *Aa* wt +Fe biofilms  *Aa* wt -Fe biofilms  *Aa* in healthy plaque  *Aa* in diseased plaque |
| S3 | Normalized expression | *Aa* wt +Fe biofilms  *Aa* *𝛥fur* +Fe biofilms  *Aa* mono-abscess  *Aa* + *Sg* co-abscess  *Aa* in healthy plaque  *Aa* in diseased plaque |
| S4 | Differential gene expression | *Aa* in diseased plaque vs. *Aa* in healthy plaque |

**Table 3. Summary of RNA-seq and ChIP-seq results.**

| Experiment | Regulation | % genome | Total | Coding | sRNA | asRNA | CRISPR |
| --- | --- | --- | --- | --- | --- | --- | --- |
| RNA-seq (low Fe vs. high Fe) | Downregulated  by low Fe | 1.3 | 30 | 30 | 0 | 0 | 0 |
|  | Upregulated  by low Fe | 2.6 | 63 | 61 | 2 | 0 | 0 |
| RNA-seq (*𝛥fur* vs. wt) | Repressed by Fur | 7.8 | 168 | 159 | 7 | 2 | 0 |
|  | Activated by Fur | 9.4 | 218 | 207 | 6 | 3 | 2 |
| ChIP-seq (Fur-VSV-G) | Directly regulated  (without operons) | 3.1 | 77 | 72 | 5 | 0 | 0 |
|  | Directly regulated  (with operons) | 3.5 | 91 | 86 | 5 | 0 | 0 |
| RNA-seq (abscess co- vs. mono-infection) | Downregulated in co-infection | 1.0 | 23 | 23 | 0 | 0 | 0 |
|  | Upregulated in co-infection | 3.6 | 83 | 77 | 4 | 2 | 0 |
| RNA-seq (oral disease vs. health) | Downregulated in periodontitis | 3.8 | 97 | 89 | 1 | 7 | 0 |
|  | Upregulated in periodontitis | 6.3 | 134 | 131 | 3 | 0 | 0 |

**Table 4. Overlap between the *𝛥fur* regulon and the low-iron and CRP regulons.**

| Overlap | Downregulated  by low iron (30) | Upregulated  by low iron (63) | Repressed  by CRP (130) | Activated  by CRP (82) |
| --- | --- | --- | --- | --- |
| Repressed  by Fur (168) | 2 | 42 | 13 | 4 |
| Activated  by Fur (218) | 19 | 7 | 38 | 18 |

Numbers in parentheses are the total number of genes in the indicated regulon. Numbers not in parentheses are the number of overlapping genes between the two indicated regulons.

**Table 5. Genes preferentially bound by Fur in the presence or absence of iron.**

| Binding | Locus tag | Gene product |
| --- | --- | --- |
| Bound in the presence of iron | 624_0115 | aminoacrylate peracid reductase |
|  | 624_0375 | hypothetical protein |
|  | 624_0433 | 5-hydroxyisourate hydrolase |
|  | 624_0772 | C4-dicarboxylate ABC transporter permease |
|  | 624_1354 | ABC transporter permease |
|  | 624_1377 | GDP-mannose dehydratase |
|  | 624_1733 | iron ABC transporter |
| Bound in the absence of iron | 624_0503 | type I-F CRISPR-associated protein Csy1 |
|  | 624_1609 | hypoxanthine phosphoribosyltransferase |
|  | 624_2476 | malonic semialdehyde reductase |
| Bound in the abscess | 624_0135 | flavodoxin FldA |
|  | 624_0136 | Fur family transcriptional regulator |
|  | 624_0137 | DNA gyrase subunit A |
|  | 624_0222 | sRNA |
|  | 624_0371 | hypothetical protein |
|  | 624_0374 | hypothetical protein |
|  | 624_0375 | hypothetical protein |
|  | 624_0376 | hypothetical protein |
|  | 624_0466 | 3-phosphoshikimate 1-carboxyvinyltransferase |
|  | 624_1008 | hypothetical protein |
|  | 624_1233 | energy transducer TonB |
|  | 624_1354 | ABC transporter permease |
|  | 624_1376 | acetyltransferase |
|  | 624_1377 | GDP-mannose dehydratase |
|  | 624_1378 | glycosyl transferase |
|  | 624_1733 | iron ABC transporter |
|  | 624_1983 | hypothetical protein |
|  | 624_2100 | hypothetical protein |

Gene promoters bound both in the abscess and preferentially in the presence of iron are highlighted in gray.

**Table 6. Overlap between the Fur-VSV-G regulon and the low-iron and *𝛥fur* regulons.**

| Overlap | Downregulated  by low iron (30) | Upregulated  by low iron (63) | Repressed  by Fur (168) | Activated  by Fur (218) |
| --- | --- | --- | --- | --- |
| Regulated by  Fur directly (91) | 2 | 32 | 33 | 7 |

Numbers in parentheses are the total number of genes in the indicated regulon. Numbers not in parentheses are the number of overlapping genes between the two indicated regulons.

**Table 7. Overlap between the co-infection regulon and the low-iron and *𝛥fur* regulons.**

| Overlap | Downregulated  by low iron (30) | Upregulated  by low iron (63) | Repressed  by Fur (168) | Activated  by Fur (218) |
| --- | --- | --- | --- | --- |
| Downregulated in co-culture (23) | 1 | 0 | 3 | 1 |
| Upregulated in co-culture (83) | 2 | 8* | 13* | 10 |

Numbers in parentheses are the total number of genes in the indicated regulon. Numbers not in parentheses are the number of overlapping genes between the two indicated regulons. *, P < 0.05 (one-tailed Fisher’s exact test).

**Table 8. *S. gordonii* genes related to iron homeostasis are not differentially expressed between abscess mono- and co-infection with *A. actinomycetemcomitans*.**

| Locus tag | Gene product | Gene category | FC  (log_2_) | P value  (adjusted) |
| --- | --- | --- | --- | --- |
| SGO_0665 | non-heme Fe-containing ferritin | DNA protection | 1.6 | 3.1E-02 |
| SGO_0703 | ferric transport regulator protein | regulation | 0.3 | 9.1E-01 |
| SGO_0767 | iron transport protein | Fe transport | 0.2 | 9.3E-01 |
| SGO_0769 | iron compound ABC transporter |  | 0.1 | 9.7E-01 |
| SGO_0770 | FecCD transport family |  | 0.1 | 9.7E-01 |
| SGO_1163 | iron permease FTR1 family |  | 0.4 | 8.7E-01 |
| SGO_1658 | ABC transporter ATP binding protein |  | 0.1 | NA |
| SGO_1311 | ferrochelatase | heme metabolism | -0.4 | NA |
| SGO_0696 | hemolysin-like protein | hemolysin | 0.6 | 7.0E-01 |
| SGO_1309 | hemolysin III-like protein |  | 0.5 | 7.9E-01 |
| SGO_1655 | possible hemolysin |  | 0.6 | 7.1E-01 |
| SGO_1029 | ferredoxin | iron sulfur metabolism | -0.8 | 6.0E-01 |
| SGO_0019 | serine dehydratase (FeS-dependent) |  | -0.4 | 8.7E-01 |
| SGO_0020 | serine dehydratase (FeS-dependent) |  | -2.6 | 1.8E-04 |
| SGO_1387 | FeS assembly protein SufB |  | -0.3 | 8.9E-01 |
| SGO_1718 | FeS assembly protein SufB |  | 0.5 | 7.6E-01 |
| SGO_1719 | SUF system FeS assembly protein |  | 0.3 | 9.2E-01 |
| SGO_1721 | FeS assembly protein SufD |  | 0.4 | 8.5E-01 |
| SGO_1722 | FeS assembly ATPase SufC |  | 0.2 | 9.2E-01 |

Highlighted fold changes (FC) are statistically significant (adjusted P value < 0.05).

**Table 9. Overlap between the periodontitis regulon and the low-iron and *𝛥fur* regulons.**

| Overlap | Downregulated  by low iron (30) | Upregulated  by low iron (63) | Repressed  by Fur (168) | Activated  by Fur (218) |
| --- | --- | --- | --- | --- |
| Downregulated in periodontitis (97) | 1 | 0 | 7 | 15* |
| Upregulated in periodontitis (134) | 1 | 11* | 17* | 13 |

Numbers in parentheses are the total number of genes in the indicated regulon. Numbers not in parentheses are the number of overlapping genes between the two indicated regulons. *, P < 0.05 (one-tailed Fisher’s exact test).

**Table 10. Summary of RNA-seq data analysis.**

| Strain | Condition | Replicate | Total reads | Processed^a^ | Mapped^b^ |
| --- | --- | --- | --- | --- | --- |
| wild type | Fe+ biofilm | 1 | 19,201,707 | 18,835,776 (98.1%) | 7,605,531 (40.4%) |
|  |  | 2 | 23,724,827 | 23,209,638 (97.8%) | 8,533,358 (36.8%) |
|  | Fe- biofilm | 1 | 12,263,466 | 11,835,032 (96.5%) | 5,692,245 (48.1%) |
|  |  | 2 | 20,503,765 | 19,949,837 (97.3%) | 7,972,082 (40%) |
| *𝛥fur* | Fe+ biofilm | 1 | 16,031,618 | 15,828,260 (98.7%) | 6,163,107 (38.9%) |
|  |  | 2 | 14,712,405 | 14,373,420 (97.7%) | 5,479,871 (38.1%) |
|  | Fe- biofilm | 1 | 19,545,394 | 19,198,805 (98.2%) | 7,725,350 (40.2%) |
|  |  | 2 | 12,692,542 | 12,400,013 (97.7%) | 5,234,624 (42.2%) |
| murine abscess  *Aa* mono-infection^c^ | | 1 | 25,297,104 | 23,449,730 (92.7%) | 2,220,474  (9.5%) |
|  |  | 2 | 42,804,092 | 40,637,742 (94.9%) | 5,094,873  (12.5%) |
|  |  | 3 | 52,165,722 | 50,487,703 (96.8%) | 6,590,138  (13.5%) |
| murine abscess  *Sg* mono-infection | | 1 | 43,522,548 | 41,142,605  (94.5%) | 593,283  (1.4%) |
|  |  | 2 | 51,137,758 | 49,318,575 (96.4%) | 517,150  (1.0%) |
| murine abscess  co-infection (*Aa*) | | 1 | 46,320,565 | 44,909,254 (97.0%) | 5,381,770 (12.0%) |
|  |  | 2 | 47,286,588 | 44,323,362 (93.7%) | 1,788,094 (4.0%) |
| murine abscess  co-infection (*Sg*) | | 1 | 46,320,565 | 44,909,254 (97.0%) | 664,529  (1.5%) |
|  |  | 2 | 47,286,588 | 44,323,362 (93.7%) | 1,416,410  (3.2%) |
| healthy human  gingival crevice^d^ | | patient 1 | 90,831,243 | 89,238,503 (98.2%) | 84,562  (0.1%) |
|  |  | patient 2 | 72,454,286 | 71,070,276 (98.1) | 68,219  (0.1%) |
|  |  | patient 3 | 156,541,929 | 155,959,986 (99.6%) | 2,000,582 (1.3%) |
| diseased human  gingival crevice^d^ | | patient 1 | 113,647,258 | 113,141,551 (99.6%) | 911,619  (0.8%) |
|  |  | patient 2 | 160,583,365 | 160,025,395 (99.7%) | 1,657,222  (1.0%) |
|  |  | patient 3 | 179,248,020 | 178,324,290 (99.5%) | 1,164,930  (0.7%) |

Percentages are relative to the left adjacent column.

^a^Reads remaining after removing low-quality bases, adaptors, and short reads

^b^Reads mapping with high quality to the reference genome

^c^See Jorth *et al.*, 2013 for sample descriptions

^d^See Jorth *et al.*, 2014 for sample descriptions

**Table 11. Summary of ChIP-seq data analysis.**

| Condition | Replicate | Total reads | Processed^a^ | Mapped^b^ |
| --- | --- | --- | --- | --- |
| Fe+ biofilm | 1 | 22,578,553 | 22,389,059 (99.2%) | 3,461,092 (15.5%) |
|  | 2 | 4,727,529 | 4,661,632 (98.6%) | 3,595,053 (77.1%) |
| Fe- biofilm | 1 | 3,968,752 | 3,935,308 (99.2%) | 1,895,819 (48.2%) |
|  | 2 | 4,312,558 | 4,301,929 (99.8%) | 3,510,204 (81.6%) |
| abscess | 1 | 17,635,818 | 17,597,007 (99.8%) | 536,151  (3%) |
|  | 2 | 7,962,573 | 7,131,965 (89.6%) | 1,849,314 (25.9%) |
| input | *in vitro* | 4,646,013 | 4,633,989 (99.7%) | 3,595,200 (77.6%) |

Percentages are relative to the left adjacent column.

^a^Reads remaining after removing low-quality bases, adaptors, and short reads

^b^Reads mapping with high quality to the reference genome

**Table 12. Summary of peak calling analysis.**

| Condition | Replicate | peaks |
| --- | --- | --- |
| Fe+ biofilm | 1 | 141 |
|  | 2 | 84 |
| Fe- biofilm | 1 | 62 |
|  | 2 | 119 |
| abscess | 1 | 138* |
|  | 2 | 67 |

*Filtered (with the MOSAiCS filterPeaks function) to remove false positives
