# Supplementary material for: Microbial Community Composition Impacts Pathogen Iron Availability during Polymicrobial Infection
Source: PLoS Pathog. 2016 Dec 14;12(12):e1006084. doi: 10.1371/journal.ppat.1006084 (PMC5156373; doi:10.1371/journal.ppat.1006084)
Supplement: S1 Table — (DOCX) [file ppat.1006084.s010.docx]

**Table S1. Cellular processes controlled by Fur.**

| Category | Fur-activated | Fur-repressed |
| --- | --- | --- |
| Central metabolism | Glycolysis (GAPDH) | Zn-dependent alcohol dehydrogenase |
|  | Pentose phosphate pathway (transketolase) | D-lactate dehydrogenase |
|  | Pyruvate formate lyase |  |
|  | Formate efflux |  |
|  | Formate hydrogen lyase |  |
|  | Reductive TCA cycle, carboxylate transporters |  |
| Respiration | Na^+^-translocating NADH-quinone reductase | Cytochrome bd biosynthesis |
|  | Nitrate reductase |  |
|  | Nitrite reductase |  |
|  | Trimethylamine N-oxide reductase |  |
|  | Dimethyl sulfoxide reductase |  |
|  | Fumarate reductase |  |
|  | Cytochrome c peroxidase |  |
| Carbon sources | Glycogen biosynthesis |  |
|  | Fructose transport, degradation |  |
|  | Galactose transport |  |
|  | Mannose transport, degradation |  |
|  | Maltose transport, degradation |  |
|  | Ribose transport, degradation |  |
|  | Mannitol transport |  |
|  | Ascorbate transport, degradation |  |
|  | Citrate degradation |  |
|  | Gluconate transport, degradation |  |
|  | Glycerol transport, degradation |  |
|  | Inositol transport, degradation |  |
| Amino acids | Tyrosine degradation | Chorismate biosynthesis |
|  | Serine transport, degradation | Serine biosynthesis |
|  |  | Valine, Leucine, Isoleucine biosynthesis |
|  | S-adenosyl-methionine biosynthesis | Homoserine metabolism |
|  |  | Alanine/glycine transport |
|  | Aspartate/asparagine biosynthesis | Aspartate/fumarate biosynthesis |
|  | Arginine biosynthesis | Arginine biosynthesis |
|  | Proline biosynthesis |  |
| Vitamins and cofactors | Biotin biosynthesis | Menaquinone biosynthesis |
|  | Pantothenate transport | Heme transport, biosynthesis |
|  |  | Folate metabolism |
| Metals | Nickel transport | Cobalt transport |
|  |  | Potassium transport |
|  |  | Chloride transport |
| Autoinducer-2 | Autoinducer-2 transport |  |
| Toxins | Leukotoxin | Cytolethal distending toxin |
| Attachment | Tight adherence | Dispersin B |
| Oxidative stress | Glutathione metabolism | Glutathione metabolism |
|  | Cytochrome c peroxidase |  |
|  | Peroxiredoxin |  |
|  | Superoxide dismutase |  |
| Membrane |  | Cardiolipin/phosphatidyl-ethanolamine biosynthesis |
|  |  | Peptidoglycan biosynthesis |
