# Supplementary material for: Microbial Community Composition Impacts Pathogen Iron Availability during Polymicrobial Infection
Source: PLoS Pathog. 2016 Dec 14;12(12):e1006084. doi: 10.1371/journal.ppat.1006084 (PMC5156373; doi:10.1371/journal.ppat.1006084)
Supplement: S2 Table — (DOCX) [file ppat.1006084.s011.docx]

**Table S2. Primer sequences.**

| Strain | Name | Sequence | Underline |
| --- | --- | --- | --- |
| *𝛥fur* | fur-ko-up-F | AAGTGCGGTTTTGTAATTCGTGCTATCACG | USS^a^ |
|  | fur-ko-up-R | CATGTATTCACGAACGAAAATCG  GGGTTCGGTAATTTTTAATCCTAC | Overlap^b^ |
|  | fur-ko-dn-F | GAAAACAATAAACCCTTGCATATG  GCACTTTTCGCCTATAAACC | Overlap^b^ |
|  | fur-ko-dn-R | AAGTGCGGTCGTCTTTGTCGGATTCATCG | USS^a^ |
|  | spec-F | CGATTTTCGTTCGTGAATACATG |  |
|  | spec-R | CATATGCAAGGGTTTATTGTTTTC |  |
| ChIP-seq | fur-pro-1-F | AAGTGCGGTCCGTTTCGAGTTAATCGTAACAG | USS^a^ |
|  | fur-tag-1-R | TTATTTACCTAAACGATTCATTTCAATATCGGTATAACC  TTTTTTGTTATTTTCTGCGCAATG | VSV-G^c^ |
|  | fur-pro-2-F | GGGGTACCCCAGTGACACAGGCAATACG | KpnI^d^ |
|  | fur-tag-2-R | GGGGTACCCC  TTATTTACCTAAACGATTCATTTCAATATC | KpnI^d^ |

^a^*A. actinomycetemcomitans* uptake signal sequence

^b^Overlap extension sequence for fusion to *aad9*

^c^VSV-G coding sequence

^d^KpnI restriction siteBottom of Form
